# Targeting MarA N‐terminal domain dynamics to prevent DNA binding

**DOI:** 10.1002/pro.5258

**Published:** 2024-12-11

**Authors:** Marina Corbella, Cátia Moreira, Roberto Bello‐Madruga, Marc Torrent Burgas, Shina C. L. Kamerlin, Jessica M. A. Blair, Enea Sancho‐Vaello

**Affiliations:** ^1^ Science for Life Laboratory, Department of Chemistry‐BMC Uppsala University Uppsala Sweden; ^2^ Departament de Química Inorgànica i Orgànica (Secció de Química Orgànica) & Institut de Química Teòrica i Computacional (IQTCUB) Universitat de Barcelona Barcelona Spain; ^3^ Department of Biochemistry and Molecular Biology Universitat Autònoma de Barcelona Cerdanyola del Vallès Spain; ^4^ School of Chemistry and Biochemistry Georgia Institute of Technology Atlanta Georgia USA; ^5^ College of Medicine and Health, Department of Microbes, Infection and Microbiomes Institute of Microbiology and Infection, University of Birmingham Birmingham UK

**Keywords:** AraC/XylS family, efflux pump regulation, MarA, mechanism of inhibition, resistance‐nodulation‐division (RND) superfamily, rob

## Abstract

Efflux is one of the mechanisms employed by Gram‐negative bacteria to become resistant to routinely used antibiotics. The inhibition of efflux by targeting their regulators is a promising strategy to re‐sensitize bacterial pathogens to antibiotics. AcrAB–TolC is the main resistance‐nodulation‐division efflux pump in Enterobacteriaceae. MarA is an AraC/XylS family global regulator that regulates more than 40 genes related to the antimicrobial resistance phenotype, including *acrAB*. The aim of this work was to understand the role of the N‐terminal helix of MarA in the mechanism of DNA binding. An N‐terminal deletion of MarA showed that the N‐terminal helix is critical for recognition of the functional marboxes. By engineering two double cysteine variants of MarA that form a disulfide bond between the N‐terminal helix and the hydrophobic core of one of the helices in direct DNA contact, and combining in vitro electrophoretic mobility assays, in vivo measurements of *acrAB* transcription using a GFP reporter system, and molecular dynamic simulations, it was shown that the immobilization of the N‐terminal helix of MarA prevents binding to DNA. This inhibited conformation seems to be universal for the monomeric members of the AraC/XylS family, as suggested by additional molecular dynamics simulations of the two‐domain protein Rob. These results point to the N‐terminal helix of the AraC/XylS family monomeric regulators as a promising target for the development of inhibitors.

## 1 INTRODUCTION

Antimicrobial resistance (AMR) is a silent pandemic with 1.27 million deaths directly attributable to resistance in 2019 (Antimicrobial Resistance Collaborators 2022; Laxminarayan 2022). In light of this scenario, urgent action is required including basic research to increase the understanding of mechanisms underpinning antibiotic. One of the mechanisms employed by Gram‐negative bacteria to become resistant to routinely used antibiotics is the use of efflux pumps to export antimicrobials out of bacterial cells (Darby et al. 2022). These efflux pumps primarily allow the microorganisms to regulate their internal environment by removing toxic substances, but other physiological roles have also been proposed, including virulence and bacterial biofilm formation (Alav et al. 2018; Henderson et al. 2021). There are seven families of efflux pumps, with the members of the resistance‐nodulation‐division (RND) superfamily being the most clinically associated with MDR phenotypes (Colclough et al. 2020; Henderson et al. 2021; Saier Jr et al. 2006). RND efflux pumps form a tripartite assembly consisting of a trimeric outer membrane protein channel, a hexameric periplasmic adaptor protein (PAP) and a trimeric inner membrane protein (Alav et al. 2021; Colclough et al. 2020; Du et al. 2014; Janganan et al. 2011; Kobylka et al. 2020; Wang et al. 2017). Members of the RND superfamily include *Escherichia coli* AcrAB–TolC, MexAB–OprM in *Pseudomonas*, MtrCDE in *Neisseria gonorrhoeae*, and *Acinetobacter* Ade systems (AdeABC and AdeIJK) (Alav et al. 2021; Colclough et al. 2020; Hagman et al. 1995; Klenotic et al. 2021). Overexpression of RND efflux pumps by mutations in their regulatory genes have been found in clinically relevant antibiotic resistance isolates (Piddock 2006).

AcrAB–TolC is the main RND efflux pump in *E. coli*. The regulation of the expression of *E. coli* AcrAB–TolC is complex and includes local regulators such as the repressors AcrR, EnvR or the activator SdiA, and global regulators such as MarA, SoxS, and Rob, which increase the expression of the major efflux genes (Barbosa and Levy 2000; Cohen et al. 1993; Weston et al. 2018). MarA, SoxS, and Rob share a high percentage of identity and similarity (e.g., SoxS and MarA share 41% identity and 67% similarity; Duval and Lister 2013) being able to bind the same 20‐bp highly degenerate DNA sequence, called the marbox. By binding to different marboxes, global regulators such as MarA can regulate 40 other genes besides *acrAB*, many of them related to the antimicrobial resistance phenotype (Barbosa and Levy 2000; Cohen et al. 1993; Weston et al. 2018).

The effectivity of *acrAB* regulation relies on the accurate balance between repression and activation. MarA is encoded by the second gene in the *marRAB* operon (Cohen et al. 1993). This operon is repressed by the dimeric MarR which binds to two specific palindromic sequences located in the promoter region *marO* (Seoane and Levy 1995). When a ligand, such as sodium salicylate, binds MarR, it releases the DNA and *marA* is transcribed activating or repressing the transcription of the genes under its control (Alekshun and Levy 1999; Duval et al. 2013). MarA also controls the *marRAB* expression by binding as a monomer to the marbox located in the promoter region of the *marRAB* operon (Martin et al. 1996). When the signal disappears, MarR binds the DNA again and the repression resumes. This mechanism coexists with other mechanisms including ligand‐triggered conformational changes in AcrR, a local *acrAB* repressor. These changes lead to the release of AcrR from the acrAB promoter, allowing transcription of acrAB (Routh et al. 2009). As shown here, the interplay between *acrAB* regulators, such as AcrR and MarA, play an important role in controlling the efflux pump expression.

**FIGURE 1:**
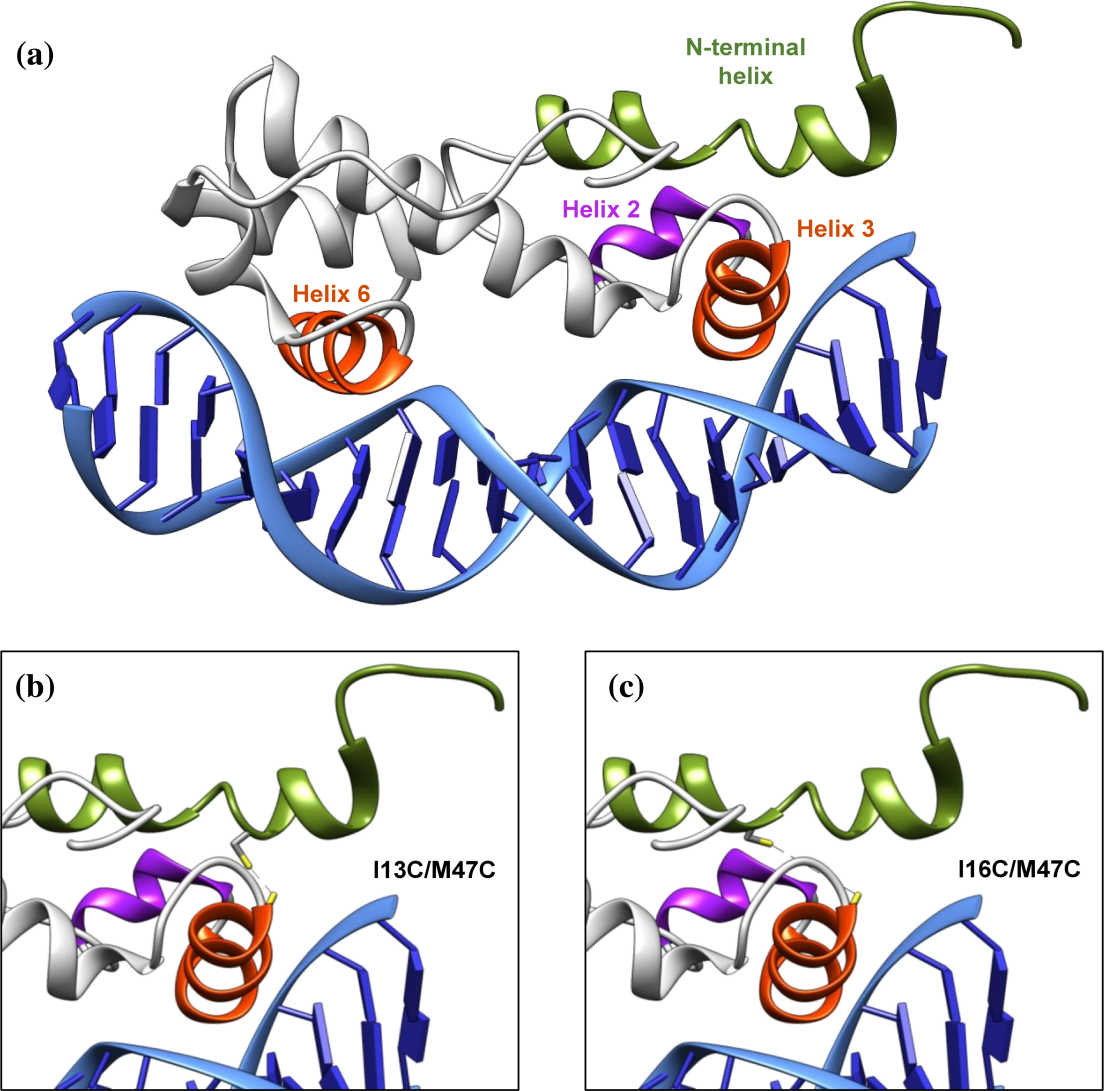
Structure of the WT MarA and its double cysteine variants used in this work. (a) MarA (PDB 1XS9) (Burley et al. 2021; Dangi et al. 2004) is a monomer with two helix–turn–helix motifs. Helices 3 and 6 (in orange) interact directly with the major groove of the DNA. The N‐terminal helix is shown in green. (b, c) Predicted structural models for the I13C/M47C and I16C/M47C variants. The formation of the disulfide bond in (b) the I13C/M47C variant maintains the N‐terminal helix in the same position as observed in the PDBs, (c) while in I16C/M47C pulls it to a different position.

The *marA* gene encodes MarA, a monomeric protein exclusively composed of α‐helices (Figure 1a). Helices 1–3 and 5–7 form the N‐terminal and C‐terminal helix–turn–helix (HTH) domains, respectively, with helix 3 and 6 being in direct contact with the DNA (Dangi et al. 2001; Dangi et al. 2004; Rhee et al. 1998). In the crystal structure, MarA bends the DNA by 35° to permit both HTH motifs to insert into the major groove simultaneously (Rhee et al. 1998). Several studies have suggested an important role for the N‐terminal helix of MarA. By NMR, it was shown that the start of the helix is close to the DNA, suggesting that the N‐terminal could contact the DNA backbone or help in the correct positioning of the helix 3 in the major groove (Dangi et al. 2001). By mutagenesis, it was shown that the N‐terminal helix was important for DNA binding and promoter activation (Gillette et al. 2000; Griffith and Wolf Jr 2002; Shah and Wolf Jr 2006). Molecular dynamic simulations showed that the flexibility of half of the N‐terminal HTH motif dominates the motions of both MarA and Rob, showing the importance of the first nucleotides of the marbox (called A‐box) for tight binding (Corbella et al. 2021; Kwon et al. 2000). In addition, the coexistence of meta‐stable forms of MarA in solution or forming complexes with DNA has been shown (Dangi et al. 2001; Koulechova et al. 2015). As it was shown that MarA exhibits multiple energetically similar conformations, it was postulated that residues distant from the active site can alter binding affinity and ligand specificity (Koulechova et al. 2015).

Many compounds that inhibit the action of efflux pumps have been discovered, but the use of many is limited by their toxicity to human cells (Marshall et al. 2020). An alternative strategy would be to target the regulators of the efflux pumps to prevent transcription of the pump genes. To date, there are no inhibitors for monomeric regulators belonging to the AraC/XylS family, such as MarA. The aim of this work is to understand the role of the N‐terminal helix of MarA in the mechanism of DNA binding. An N‐terminal deletion of MarA showed that the N‐terminal helix has a role in the recognition of the functional marboxes. By engineering two double cysteine variants of MarA, it was shown that the immobilization of the N‐terminal helix of MarA prevents binding to DNA. This inhibited conformation seems to be universal for the monomeric members of the AraC/XylS family, as suggested by complementary molecular dynamics simulations of the two‐domain protein Rob. These results point to the N‐terminal helix of the AraC/XylS family monomeric regulators as a promising target for the development of inhibitors.

## 2 RESULTS

### 2.1 The N‐terminal helix of MarA is a crucial structural element for the mechanism of DNA binding

MarA is a monomeric protein composed of two helix–turn–helix (HTH) domains, with helices 3 and 6 making direct contact with the DNA (Figure 1a). Even though the N‐terminal helix of MarA does not directly contact the DNA in the crystal structures, previous work, including NMR and mutagenesis studies, suggested that this structural element could be involved in DNA binding, affecting to different extents the activation of the promoters under its control (Dangi et al. 2001; Gillette et al. 2000; Griffith and Wolf Jr 2002; Shah and Wolf Jr 2006). Also, the coexistence of distinct doubling resonances affecting the first 10 residues of MarA indicated the coexistence of two conformations for this region of the protein (Dangi et al. 2001).

In order to check the importance of the free movement of the N‐terminal helix of MarA in the mechanism of DNA binding, we constructed two double cysteine variants to block the movement of this structural element through the formation of a disulfide bond (Figure 1). One of the variants, I13C/M47C, was designed to maintain the N‐terminal helix in the same position as observed in the published MarA structures (Cα–Cα distance of 7 Å in the wild‐type MarA crystal structure (PDB 1BL0 and 1XS9) (Burley et al. 2021; Dangi et al. 2004; Rhee et al. 1998)) (Figure 1b). In the other variant, I16C/M47C, the formation of the disulfide bond immobilizes the N‐terminal helix outside the position observed in the PDBs (Cα–Cα distance of 11 Å in the wild‐type MarA crystal structure) (Figure 1c). This is a direct consequence of the longer distance between the two cysteines introduced at positions 16 and 47.

Wild‐type (WT) MarA and its variants were overexpressed, purified, and tested in an electrophoretic mobility shift assay (EMSA) by using 200‐bp DNA fragments harboring the sequence of the marbox of the *marRAB* operon centered in the fragment. These experiments were performed in the presence and absence of dithiothreitol (DTT) which reduces the cysteines and breaks the disulfide bond, releasing the N‐terminal helix of MarA and allowing its free movement.

**FIGURE 2:**
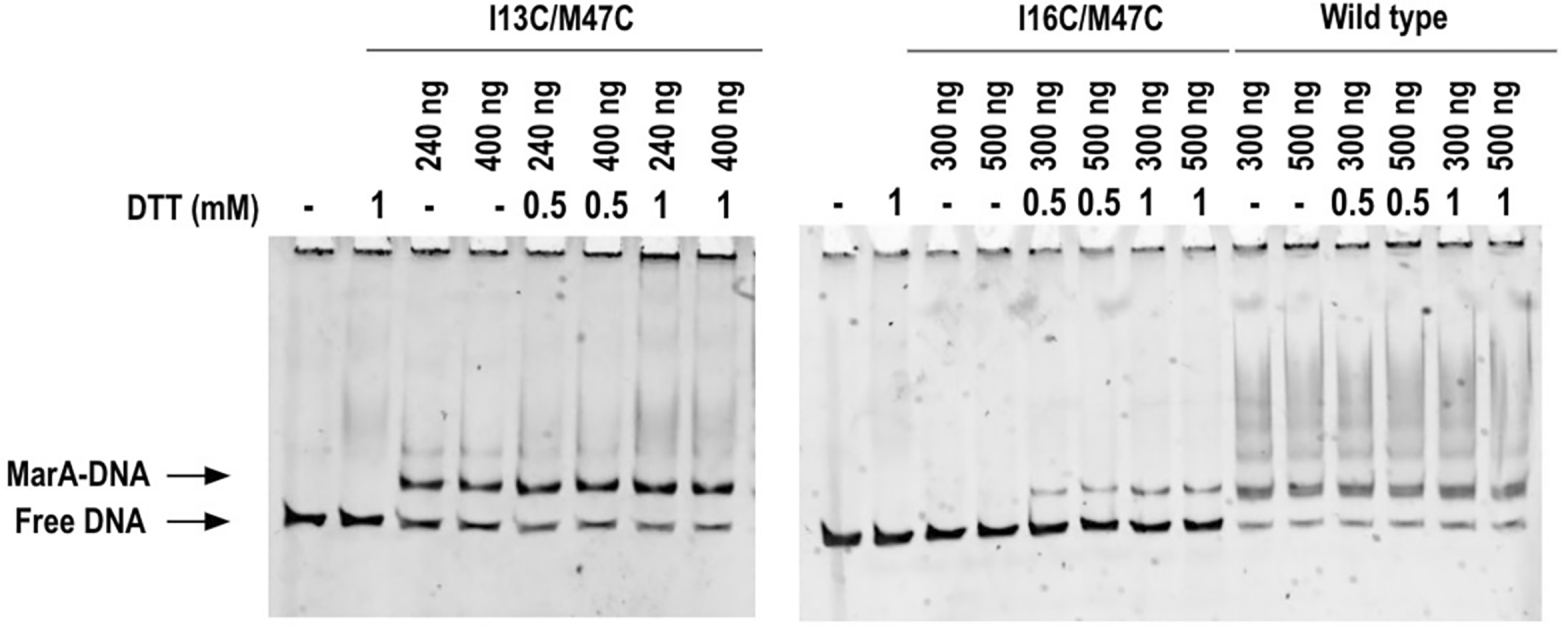
EMSA for the WT MarA and its double cysteine variants I13C/M47C and I16C/M47C in the presence and absence of DTT by using 200‐bp DNA fragments containing the *marRAB* marbox. The multiband pattern is only observed for the WT protein.

The I13C/M47C variant and WT MarA were both able to bind the marbox in the absence and presence of DTT (Figure 2). By contrast, the variant I16C/M47C was only able to bind the marbox when the DTT was present, that is, when the disulfide bond constraining the free movement of the N‐terminal helix was broken (Figure 2). The same pattern was observed for WT, I13C/M47C, and I16C/M47C when using 200‐bp DNA fragments harboring the *acrAB* marbox or 30‐bp DNA fragments composed of the *marRAB* marboxes (Figures S1 and S2a and Table S1, Supporting Information). The binding specificity was confirmed by using a 200‐bp DNA fragment that does not include any marbox sequence as a negative control. As shown in the gels, WT MarA or its variants were not able to shift this DNA fragment (Figure S1 and Table S1). WT MarA or its variants were also unable to shift a 30‐bp non‐specific DNA fragment confirming the specificity in DNA binding for WT MarA and its variants (Figure S2a, right panel).

WT MarA is known to be unstable when it is not DNA‐complexed and it becomes more ordered upon the formation of the MarA–DNA complex (Corbella et al. 2021; Dangi et al. 2004; Rhee et al. 1998). Since the I16C/M47C variant was even more unstable that the WT (shown by the protein precipitation after overnight storage at 4°C), the EMSAs were performed with the N‐terminal His‐tagged proteins (Figure 2). To ensure that the His‐tag did not affect the EMSA results, this tag was cleaved using thrombin and the cleaved proteins were separated by affinity chromatography. During the cleavage and subsequent separation of the His‐tag and the cleaved protein, the concentration of the I16C/M47C variant dropped dramatically. Even with this, we succeed in performing the EMSA with the cleaved variants and confirmed that the pattern of bands was similar to the one observed for the His‐tagged proteins (Figure S2b and Table S1).

Strikingly, even when the I13C/M47C variant was able to bind the marboxes in both reductive and oxidative conditions, it never showed the same multiband pattern observed for wild‐type MarA (Figures 2, S1, and S2b). To know if this pattern corresponded to non‐specific binding, we performed competitive EMSAs. As the detection method used to reveal the EMSAs was fluorescence (see section 4), we could not use the common competitive EMSA approach consisting on using non‐labeled *acrAB* marbox fragments as a competitor DNA. Instead of using this approach, we performed two competitive EMSAs by using 200‐bp DNA fragments containing the *acrAB* marbox and (1) salmon sperm DNA or (2) the previously used 200‐bp non‐specific DNA fragment (Figure S1) as non‐specific competitors (Figure S3). As observed in both gels, WT MarA maintained the multiband pattern, while the I13C/M47C variant showed a single shifted band (Figure S3a,b). In this sense, the multiband pattern exhibited by WT MarA could not be related to non‐specific binding. Further research must be done to clarify this.

To ensure that the introduction of two cysteines in MarA was not affecting the EMSAs, the S62C/L121C variant was designed as a negative control for the formation of the bonds. Specifically, in S62C/L121C, the disulfide bond blocked the movement of the C‐terminal tail since the residues 62 and 121 are located in the helix 4 and the C‐terminal tail, respectively (Cα–Cα distance of 6 Å in the wild‐type MarA crystal structure) (Figure S4a). As expected, the ability to bind DNA in the presence or absence of DTT was not affected when using a 200 bp DNA fragment containing the *marRAB* sequence (Figure S4b).

The formation of the disulfide bond in the N‐terminal MarA variants was analyzed by ESI‐MS under oxidative and reductive conditions. While the wild‐type MarA lacks cysteine residues in its amino acid sequence, both variants contain the two introduced cysteines. It is well known that the formation of a disulfide bond results in a 2‐Da reduction of molecular weight, which can be identified by mass spectrometry (Tsai et al. 2013). Therefore, the 2‐Da mass variation for the I13C/M47C and I16C/M47C variants in the presence (reductive environment) and absence (oxidative environment) of 1 mM DTT would allow us to determine the oxidation state of these proteins. The measured molecular masses for WT MarA allowed us to identify the N‐terminal methionine excision, a common co‐translational modification (Figure S5a) (Marino et al. 2015). Taking this into consideration, the calculated molecular masses were in excellent agreement with the theoretical one. The average of the calculated MW was 17205.57 ± 0.87 Da and 17205.40 ± 0.70 Da in the absence and presence of 1 mM DTT, respectively (the expected MW was 17205.56 Da) (Table S2 and Figure S5b). Under oxidative conditions, the deconvoluted molecular mass from more than three peaks for I13C/M47C and I16C/M47C gave the theoretically calculated one for their respective oxidized forms (MW = 17165.44 Da) (Tables S3 and S5 and Figure S5b). Additionally, under reducing conditions, we detected ions which mass correspond to the reduced form of the protein (17167.46 Da) (Tables S4 and S6 and Figure S5b). Under oxidative and reductive conditions, we could also find monoisotopic ions corresponding to the reduced and oxidized form of the protein, respectively. This result shows that disulfide bond formation/breakage does not happen at 100%, which may be due to the strength of the bond, the stability of the reducing agent or the poor accessibility of DTT due to steric impediment in the N‐terminal region. The presence of monoisotopic ions corresponding to the oxidized form under reducing conditions can also be explained by disulfide bond rearrangement, as reported in studies with other proteins (Hustoft et al. 2012; Tsai et al. 2013). However, the predominant presence of monoisotopic ions corresponding to the respective conditions tested allowed us to confirm the existence of one intramolecular disulfide bridge in the I13C/M47C and I16C/M47C variants.

### 2.2 Constraining movement of the MarA N‐terminal helix reduces activation of acrAB transcription

In order to test the ability of the variants to bind the marbox and induce transcription in vivo, a fluorescent transcriptional reporter system was used. Specifically, it consisted of the pACYC177 plasmid containing the *E. coli acrAB* promoter followed by the gene encoding green fluorescent protein (GFP). This plasmid was named pACYC177‐RS. Since we were interested in studying the activity of the variants after formation of the disulfide bond, we used shuffle T7 express *E. coli* cells that lack important reductases located in the *E. coli* cytoplasm generating an oxidative folding that allows the formation of disulfide bonds in the cytoplasm (Bardwell et al. 1991; Lobstein et al. 2012). The pACYC177 plasmids and pET‐15b harboring WT *marA*, I16C/M47C or I17C/M47C were co‐transformed and *acrAB* expression was assessed by measuring GFP fluorescence.

**FIGURE 3:**
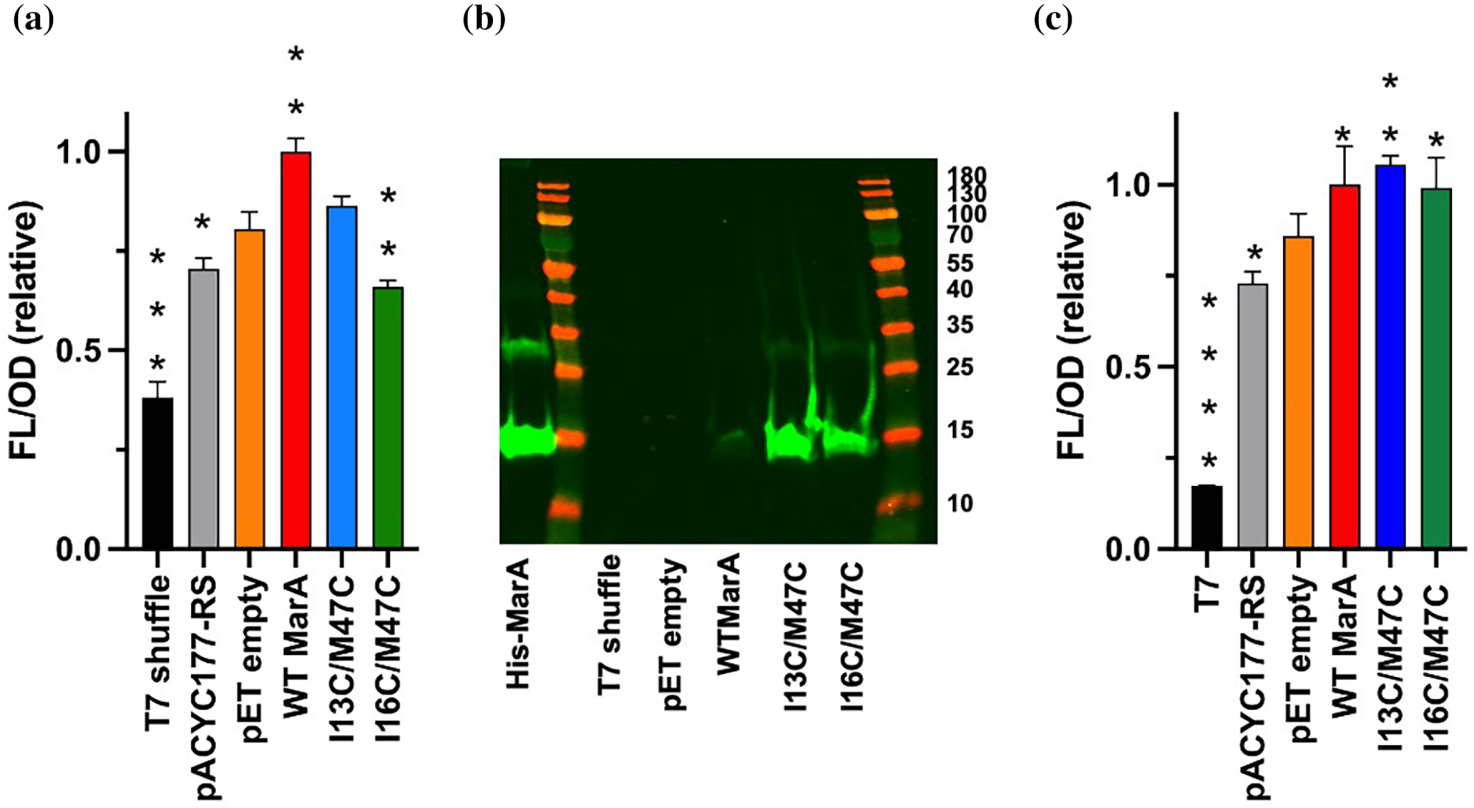
(a) Normalized fluorescence observed for shuffle T7 express cells, pACYC177‐RS, pET‐15b empty vector, WT MarA, I13C/M47C, and I16C/M47C. The decrease in fluorescence exhibited by the I16C/M47C variant is statistically significant (tested by a Student's *t* test). (b) Western blot to detect the His‐tagged MarA protein in shuffle T7 express cells, and shuffle T7 express cells harboring the pET‐15b empty vector, or pET‐15b harboring WT MarA, or I13C/M47C, or I16C/M47C. Western blotting was performed using a primary anti‐His‐tag mouse antibody and a secondary goat anti‐mouse fluorescent antibody. MarA can be detected in the cells containing WT MarA and variants, but not in the shuffle T7 express cells or pET‐15b empty vector. (c) Normalized fluorescence for the T7 express *E. coli* cells alone, the reporter system (pACYC177‐RS) or harboring both pACYC177‐RS and pET‐15b empty vector, WT *marA* gene, I13C/M47C variant, or I16C/M47C variant genes. In (a, c) fluorescence raw data were divided by OD and normalized such that WT MarA values were 1. The asterisks indicate statistically significant differences between pET‐15b empty and the rest of the strains (tested by a Student's *t* test).

When the cultures were grown to OD_600_ = 0.5 and induced with 0.5 mM IPTG for 90 min at 37°C, an increase in normalized fluorescence was observed for the cells containing the pET‐15b plasmid harboring WT MarA in comparison with the variants or pET‐15b empty vector (Figure 3a). The presence of pET‐15b (empty vector) induced the synthesis of GFP slightly. The increase of fluorescence when adding empty vectors is a well‐known effect that can be attributed to transcription initiation from cryptic promoter elements or regulatory elements present in the vector backbone (Trauth and Bischofs 2014). I13C/M47C showed an increase in the fluorescence signal showing that, even by being able to bind the DNA in the EMSAs, this variant cannot induce the transcription of the GFP protein in the reporter system as the WT MarA does. Strikingly, I16C/M47C showed a signal lower than the empty vector, suggesting that the synthesis of I16C/M47C hinders the DNA binding of the endogenous WT MarA. However, even by adding increasing concentrations of I16C/M47C to the EMSA, there was no observable decrease in the ability of the WT MarA to bind the marbox (Figure S6).

The expression of WT MarA and variants in the shuffle T7 express IPTG‐induced cultures was checked by western blotting (Figure 3b). No expression of MarA was observed in shuffle T7 express cells or harboring the pET‐15b empty vector. The signal for WT MarA was much lower than for the variants. The same result was observed when using another antibody, in this case, an HRP‐conjugated anti‐His tag antibody, to reveal the membrane (Figure S7). The low expression of WT MarA in shuffle T7 express cells is supported by a recent paper in which the authors observed by WB the decline of the signal corresponding to the His‐tagged AraC/XylS transcriptional factor Caf1R when inducing in shuffle T7 express cells at 37°C (Gahlot et al. 2021). As the fluorescence signal for WT MarA was high in the reporter system experiment (Figure 3a), we discarded the lack of overexpression of the protein and focused on its degradation. Griffith et al. identified the Lon protease as the main protease involved in SoxS and MarA degradation, although they raised the possibility that a second protease also degrades MarA with very different kinetics (Griffith et al. 2004). Shuffle T7 express *E. coli* strain is a Lon/OmpT deficient strain, so this extra protease could be acting in these cells. The WB also shows the I13C/M47C and I16C/M47C variants being less prone to degradation suggesting that the formation of the disulfide bond could avoid the degron recognition and reinforcing the idea that other non‐identified proteases can degrade WT MarA by recognition of its N‐terminal sequence (Griffith et al. 2004; Shah and Wolf Jr 2006).

In order to test in vivo if the variants can bind DNA when the disulfide bonds are not formed, we co‐transformed into T7 express *E. coli* cells both, pACYC177‐RS and pET15b empty or harboring WT MarA or the I13C/M47C or I16C/M47C genes (Figure 3c). In this cell background, the reductive cytoplasm does not allow the formation of the disulfide bonds in the MarA variants. The cultures were grown to OD_600_ = 0.5 and induced with 0.5 mM IPTG for 30 min, 37°C, and an increase in normalized fluorescence was observed for the cells containing the pET15b plasmid harboring WT MarA or its variants in comparison with pET‐15b empty vector. These results show how the I16C/M47C variant can bind the *acrAB* marbox when the disulfide bond is not formed, in agreement with the results observed in the EMSAs when adding a reducing agent.

### 2.3 Impact of the I13C/M47C and I16C/M47C double cysteine mutations on the dynamics and structure of free MarA

**FIGURE 4:**
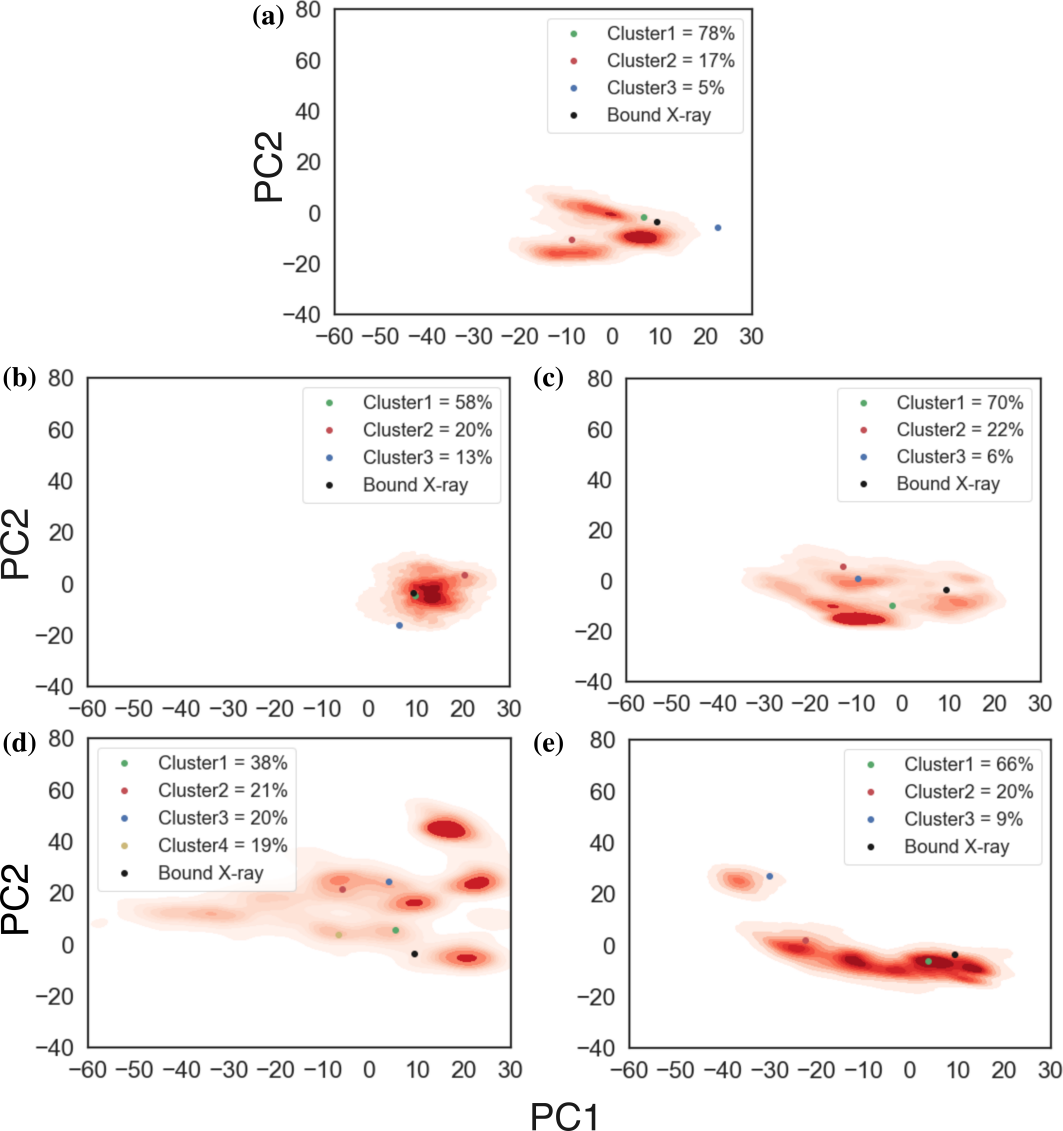
Projection of the conformational space sampled in our simulations of DNA‐free wild type and MarA variants onto the first two principal components obtained from principal component analysis (PCA) on our MD simulations. Shown here are data for simulations of (a) wild‐type MarA, (b) the MarA I13C/M47C double variant that inserts a disulfide bridge into the system, (c) the MarA I13C/M47C double variant with an artificially broken disulfide bridge (named I13C/M47C^broken^) to mimic the effect of adding dithiothreitol (DTT) to the system, (d) the MarA I16C/M47C double variant with a disulfide bridge present in the starting structure, and (e) the MarA I16C/M47C^broken^. Representative structures obtained from hierarchical agglomerative clustering on the root mean square deviation (RMSD) of the C_a_ carbon atoms, performed as described in the Methods section (Figure S11), are projected onto the two PCs as colored dots. The structure of the *mar*‐bound MarA crystal structure is represented by a black dot (PDB ID: 1BL0) (Burley et al. 2021; Rhee et al. 1998).

To assess the impact of the MarA I13C/M47C and I16C/M47C double cysteine substitutions on the dynamical and structural properties of MarA, we performed molecular dynamics (MD) simulations of both MarA variants in the absence of DNA, as described in section 4 (Figure S8a–c). As already shown in Corbella et al. (2021), while there are some differences in mobility between free MarA and the DNA‐bound crystal structure (Figures 4a and S9), the representative structure of the main cluster (containing 78% of all conformations sampled during our simulations) deviates minimally from the conformational space sampled by the DNA‐bound crystal structure, so this conformation of MarA can likely easily bind the promoter sequence. It is interesting to note that the most mobile regions, both in free MarA (Figures S9 and S10) and when complexed with the DNA (Corbella et al. 2021, fig. S42), are helix 2 and the loop connecting helix 2 and helix 3, containing residues Ser37, Gly38, and Tyr39 showing doubling of resonances in NMR studies (Dangi et al. 2001).

In the case of the MarA I13C/M47C double variant (Figure 4b), PCA analysis suggests the presence of a single large basin with focused sampling of conformational space in our simulations (more so than in simulations of the wild‐type enzyme that lacks this disulfide bridge), with representative structures from the centroid of each cluster overlaying on that of the wild type to within a C_α_‐atom RMSD of 2 Å. This suggests that the disulfide bridge already stabilizes the conformation of MarA, even in the absence of ligand, as shown in the RMSF analysis, with a reduced mobility of the N‐terminal HTH motif (residues 25–51, Figure S10). Nonetheless, when the disulfide bridge is broken, our PCA analysis shows similar mobility as observed in the wild type, although the representative structure of the main cluster slightly deviates from the conformational space sampled by the DNA‐bound crystal structure (Figure 4c). In contrast, the MarA I16C/M47C variant appears to be far more conformationally diverse, both with a formed disulfide bridge (Figure 4d) and when the disulfide bridge is artificially broken in our simulations (named I16C/M47C^broken^) (Figure 4e). That is, our PCA analysis yields multiple energy basins representing different conformations of the system, irrespective of whether the disulfide bridge is formed or not. Furthermore, the broader diversity in conformations that appear to be sampled in simulations of this variant suggest a higher likelihood to sample also unproductive conformations that are unable to bind the *mar* promoter.

Following from this, visual inspection of representative structures from the centroids of each of the clusters shown in Figure S9 shows subtle differences in structure between variants at the N‐terminal HTH motif, in particular revealing structural deviations in helices 2 and 3, that partially lose their secondary structure integrity and adopt a position that would be further away from where the promoter would be expected to bind to the protein (based on the crystal structure of the DNA‐bound transcription factor; see Figure S9). This implies that for DNA binding to occur in these variants, it would require more substantial bending of the *mar* promotor for MarA to be able to establish interaction with the A‐box region of *mar* promoter. Furthermore, secondary structure analysis using DSSP (Kabsch and Sander 1983; Touw et al. 2015) shows that while the helix 3 (residues 41–52) maintains its helicity during simulations of wild‐type MarA and the I13C/M47C double cysteine variant, the helicity is relatively stable over the course of the simulations at the N‐terminal portion (residues 41–47) but slightly lost at the C‐terminal portion (residues 48–52) (Figure S11). In simulations of the MarA I16C/M47C double cysteine variant, the percentage of helicity is slightly reduced, especially at the C‐terminal portion (residues 45–52) although still between 60% and 80%. Upon breaking the disulfide bridge to mimic the effect of DTT (Figure S11), helicity is rescued showing again same helicity as in the wild type (Figure S11). This loss of helicity could impact the ability of MarA to bind to its promotor sequence, given the importance of R46 and W42 on the helix 3 in establishing a strong binding interaction between MarA and mar promotor (Burley et al. 2021; Corbella et al. 2021; Martin et al. 1999). Nonetheless, the fact that the loss of helicity is observed in both I13C/M47C and I16C/M47C double cysteine variants, being the first fully capable of binding the mar sequence, discards a change on the helicity as the reason why I16C/M47C variant is unable to bind it. This fact reinforces the dynamical role of the N‐terminal domain.

It is worth noting that the introduction of a disulfide bridge between residues I13 and M47 does not create major structural perturbation to the MarA structure, as these residues lie in relatively close spatial proximity (Cα–Cα distance of 7 Å in the wild‐type MarA crystal structure, PDB ID: 1BL0 (Burley et al. 2021; Rhee et al. 1998)). In contrast, residues I16 and M47 are 11 Å apart using the same distance metric, and therefore enforced formation of a disulfide bridge between these residues would be expected to cause greater structural perturbation. We would like to note that despite the static distance in the crystal structure between I16 and M47 alpha carbons is 11 Å, the system is subjected to small fluctuations that can eventually bring the two alpha carbons into an acceptable threshold (3.0–7.5 Å) for a disulfide bridge formation (Gao et al. 2020) without the need for artificial displacement of the N‐terminal helix or helix 3 (Figure S12). For comparison, we therefore also performed simulations of the DNA‐free MarA I13C/M47C^broken^ and I16C/M47C^broken^ variants in which we manually broke the disulfide bridge (see section 4), in order to mimic the effect of adding DTT to the system. In the DSSP analysis (Figure S11) we observe a rescue of the wild‐type helicity in the helix 3 upon removal of the disulfide bridge, while for helix 2, the helicity is unchanged for I13C/M47C variant and a loss of helicity is observed in I16C/M47C variant upon removal of the disulfide bridge.

### 2.4 Intrinsic fluorescence measurements suggest that the disulfide bonds do not affect the overall fold of the N‐terminal domain

To ensure that the I16C/M47C variant was not prevented from binding to the DNA by an indirect distortion of the whole N‐terminal domain produced by the introduction of the disulfide bond, we performed intrinsic fluorescence measurements. WT MarA has two tryptophans in its sequence, W19 and W42, located in the N‐terminal helix and helix 3, respectively. Intrinsic fluorescence is a powerful technique that allows conformational changes in proteins to be monitored by studying the tryptophan signal which is highly dependent on the local microenvironment (Dos Santos Rodrigues et al. 2023). We reasoned that, in the absence of DTT, the maintenance of the fluorescence for W42 in the two MarA variants would indicate that the overall folding of the whole N‐terminal domain is not being affected by the formation of the disulfide bond. Two peaks around 350 nm and 380 were observed in the WT MarA spectrum (Figure S13a, red line). To determine which of the two peaks corresponded to W19, we synthesized the W42F MarA variant with only one tryptophan in its sequence. As expected, this variant only showed one of the two peaks, specifically the one around 350 nm, allowing the identification of the peak corresponding to W19 (Figure S13a, orange line). When measuring the I13C/M47C and I16C/M47C variants some differences around 350 nm were observed (Figure S13a, blue and green lines). This can be explained since I16C/M47C pulls more of the N‐terminal helix being W19 surrounded by different neighbor residues and generating a different microenvironment. A slight difference in the signal around 380 nm was also observed when comparing WT MarA and the variants which could be a consequence of the disulfide bond formation. In contrast, we could not see any difference in the signal of the peaks around 380 nm for the I13C/M47C and I16C/M47C variants, meaning that the microenvironment around them was similar. This effect can be observed in the most populated clusters of each complex, where W19 sidechain is shown in slightly different conformations. Of special relevance is the cation–π interaction observed in WT MarA between W19 and R24, which is lost in both double cysteine variants. Contrarily, the microenvironment of W42 does not seem to be affected in free MarA simulations (Figure S13b). The fact that the two variants have the same fluorescence signal at 380 nm but show different behavior in the EMSAs (the I13C/M47C and I16C/M47C variants able and unable to bind DNA, respectively) strongly suggests that the inability of I16C/M47C for binding the DNA after disulfide bond formation is not related to the distortion of the N‐terminal domain. In this way, the inability of the I16C/M47C variant to bind DNA once the disulfide bond is formed could only be explained by the restriction of the movement of the N‐terminal helix, imposed by the disulfide bond formation.

### 2.5 Impact of the I13C/M47C and I16C/M47C double cysteine variants of MarA on marbox binding

To assess how the dynamic observations made in our simulations of free MarA and the I13C/M47C and I16C/M47C double cysteine variants potentially affect marbox binding, we also performed MD simulations of both MarA variants in complex with the *mar* promoter (30 bp fragment). In all systems, MarA formed an interaction with the marbox in the starting structure of the simulations (Figure S14a). Based on these starting structures, despite performing our sampling on the μs timescale, we did not observe unbinding of the marbox from MarA on the simulation timescales used here using conventional molecular dynamics (cMD) simulations (Table S7), and therefore we used instead Gaussian‐accelerated MD simulations (GaMD) to enhance the sampling of our simulations and allow for potential transitions between bound and unbound states (Corbella et al. 2021; Miao et al. 2015).

Following from this, we observed no marbox unbinding events in our GaMD simulations of the MarA(I13C/M47C)–*mar* complex in any of the five replicates during 800 ns of simulation time (Figure S15a), in agreement with the experimental observation that this double variant is able to bind the marbox even in the absence of DTT. This is also consistent with the observation from our simulations of the corresponding DNA‐free protein, which show that this disulfide bridge restricts the conformation of the N‐terminal helix of MarA into the bound conformation observed in the crystal structure (Figure S9). In contrast, in the GaMD simulations of the MarA(I16C/M47C)–*mar* complex, three out of five replicas lost interactions with the A‐box of the *mar* promoter at the N‐terminal side where the disulfide bridge is inserted, while the B‐box stayed bound in all our simulations (Figure S15b). A‐box unbinding observations are again in line with both the experimental data and the free MarA(I16C/M47C) simulations (Figure S9), showing that this double cysteine variant prevents the N‐terminal HTH motif of MarA to properly bend to bind the *mar* promoter. It is interesting to note here that the MarA(I16C/M47C) disulfide bridge triggered A‐box unbinding but had no effect on B‐box binding, while we did previously observe that introducing mutations at the nucleobases of the A‐box provoked both A‐box and B‐box unbinding on a relatively short timescale (Corbella et al. 2021). This is interesting from a recognition mode perspective, since according to our simulations, MarA seems to not release the promoter if the sequence of A‐box of the marbox is not altered, pointing to a key role of the A‐box in the MarA recognition mechanism (Kwon et al. 2000).

**FIGURE 5:**
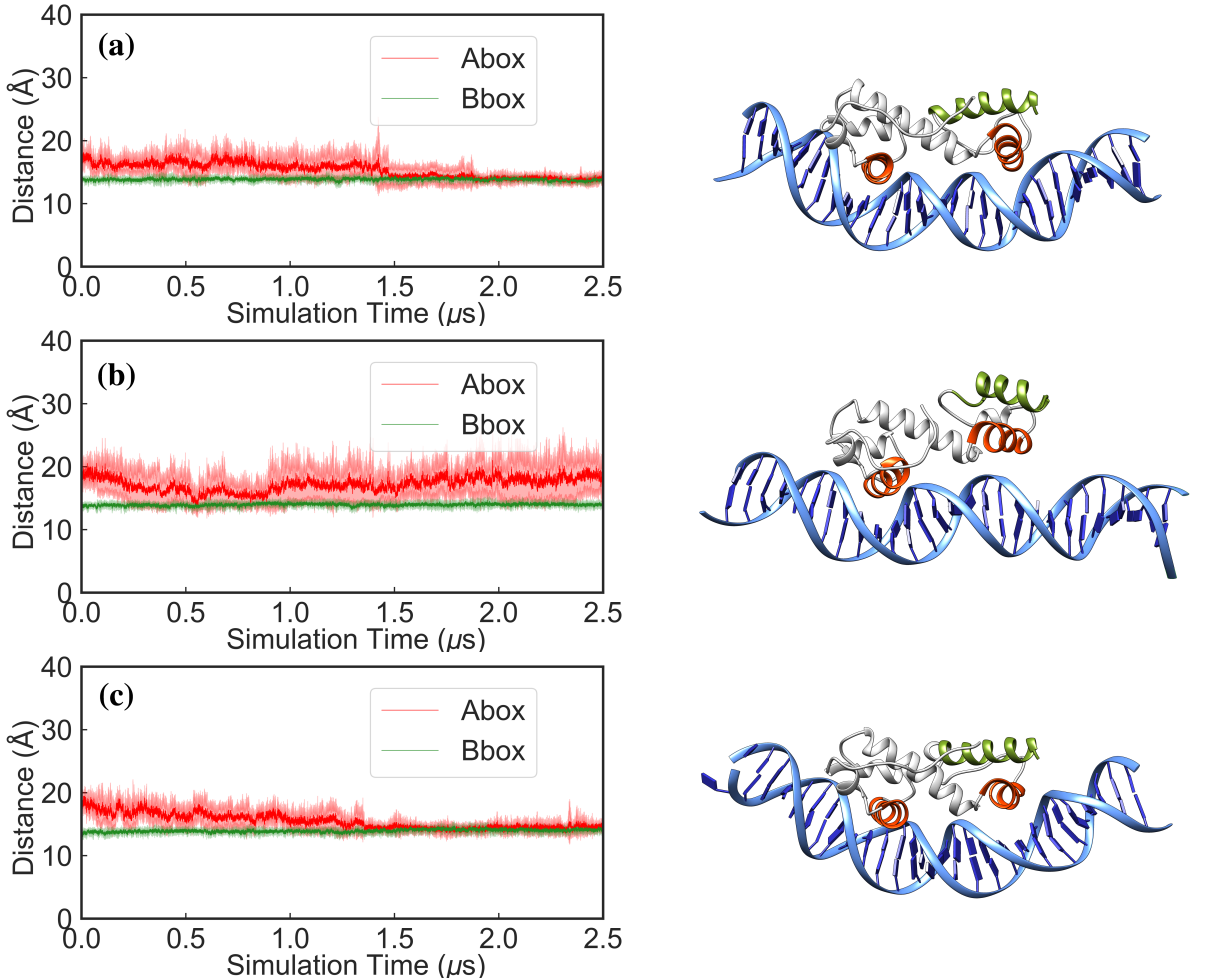
Time evolution of the distances between the helices inserted inside the major groove and the base pairs at the A‐box and B‐box (shown in red and green, respectively) during 2.5 μs of conventional MD simulations of the (a) the wild‐type MarA–*mar* complex, and (b) the MarA (I16C/M47C)–*mar* and (c) MarA(I16C/M47C)–*mar* complexes. Note that the starting point for these simulations was the A‐box unbound MarA(I16C/M47C)–*mar* complex obtained from our GaMD simulations, where in (a) the double variant was reverted to wild type and in (c) the corresponding disulfide bridge was broken by protonating each cysteine side chain. Shown here also are examples of different binding conformations during our simulations selected based on visual examination of the trajectories.

To further characterize the unbinding events observed in our GaMD simulations of the MarA(I16C/M47C)–*mar* complex, we used this partially unbound structure as a starting point for new cMD simulations (5 × 2.5 μs) of both the MarA(I16C/M47C)–*mar* complex, and a system where the double cysteine variant was reverted to wild type (Figure S8d,e). We monitored the time evolution of the insertion of both MarA HTH motifs inside the A‐box and B‐box of the *mar* promoter, tracking the distances between helices 3 and 6, which are inserted inside the major groove and the base pairs at the corresponding boxes, as we did in our previous work (Corbella et al. 2021) (i.e., between Lys41–Thr52 and nucleotides 21–22 and 39–40 for the A‐box and Gln91–Phe102 to nucleotides 12–13 and 49–50 for the B‐box; Figure 5). On the basis of this data, we observe that the MarA(I16C/M47C) is not able to insert helix 3 inside the A‐box of the *mar* promoter again, in agreement with the experimental observations, whereas the wild type reverted complex does bind the A‐box of the *mar* promoter after 1.5 μs of simulation time despite starting from an unbound conformation, and this binding interaction remains stable until the end of our simulations (2.5 μs of simulation time; Figure 5).

Finally, we aimed to mimic the addition of DTT to the MarA(I16C/M47C)–*mar* complex by using the same A‐box unbound complex from our GaMD simulations but breaking the disulfide bridge by protonating the cysteines in both 16C and 47C positions (named I16C/M47C^broken^), and again performing (5 × 2.5 μs) of cMD simulations (Figure S8f). We tracked the same distances (Figure 5c) and observed that after 1.5 μs of simulation time, MarA is again able to interact with the A‐box of the *mar* promoter, retaining a stable interaction until the end of the trajectory. Furthermore, we also performed cMD simulations (5 × 2.5 μs) using the same A‐box unbound complex from our GaMD simulations, breaking the disulfide bridge and exchanging the two cysteine residues for serine to break the linkage between them while maintaining the disulfide bridge formed conformation of MarA as a starting point (Figure S8g). Again, when tracking the same distances, we observed that after around 1 μs of simulation time, MarA is again able to bind to the A‐box of the *mar* promoter (Figure S16). This strongly supports that formation of this disulfide bridge is altering the conformational dynamics of MarA, as well as the structure of the N‐terminal HTH motif, in such a way that MarA is no longer able to bind the marbox of the *mar* promoter (Corbella et al. 2021; Shi et al. 2022), and not simply due to a perturbation effect on the hydrophobic environment.

### 2.6 The N‐terminal helix of MarA seems to be involved in the recognition of the marboxes

MarA binds a degenerate 20‐bp DNA sequence present with approximately 10,000 copies in the *E. coli* genome, most being non‐functional (Martin et al. 1999; Martin et al. 2002). The mechanism by which MarA differentiates between the functional and non‐functional marboxes remains unclear. To determine whether the N‐terminal helix of MarA is involved in this mechanism of recognition, the whole N‐terminal helix (ΔH1–MarA) was deleted. As the deletion of the N‐terminal helix exposed the hydrophobic core of the N‐terminal HTH domain, a fragment containing the His‐tag and 10 additional amino acids was placed in its place (Figure S17a). After purification of the overexpressed protein using the same protocol used for WT MarA, with some variations to optimize the obtaining of the DNA‐free ΔH1–MarA protein (see section 4), ΔH1–MarA was found forming a complex with DNA, shown by a high 260/280 ratio (260/280 = 1.5) and by a SYBR green and Coomassie double stained gel (Figure S17b). As expected, the pure ΔH1–MarA protein was not able to bind DNA in an EMSA (Figure S17c).

**FIGURE 6:**
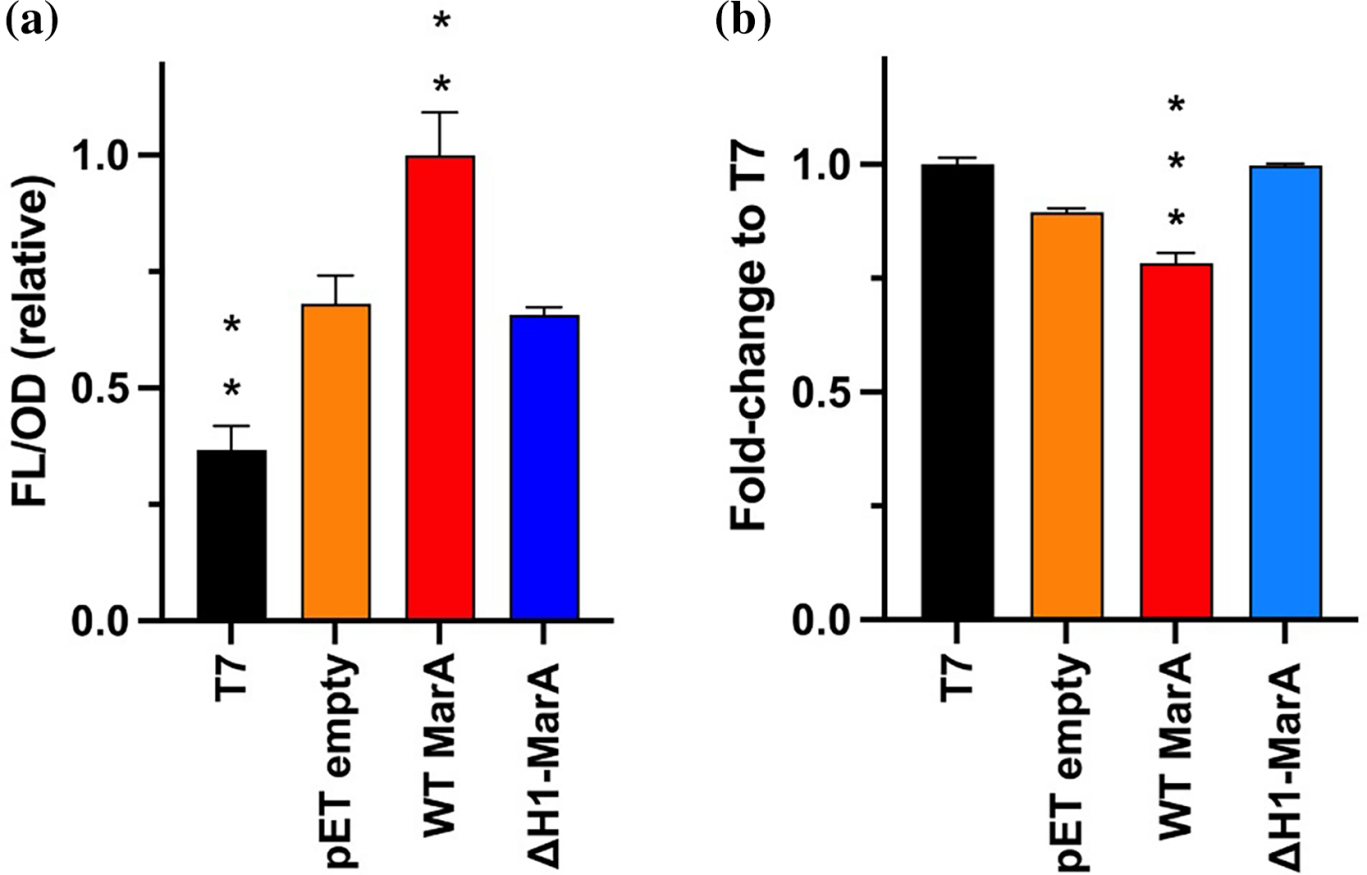
(a) The normalized fluorescence (FL/OD) in the reporter system shows an increase in signal after wild‐type (WT) MarA overexpression, but not when ΔH1–MarA is overexpressed. Fluorescence raw data were divided by OD and normalized considering WT MarA values as 1. The asterisks indicate statistically significant differences between pET empty and the rest of the strains (tested by a Student's *t* test). (b) Accumulation assay performed in T7 express *E. coli* cells shows that ΔH1–MarA has no activity in comparison with the WT MarA, which is able to induce *acrAB*–*TolC* and reduce the accumulation of the dye ethidium bromide. The decrease in accumulation exhibited by WT MarA versus T7 express cells is statistically significant (shown by a Student's *t* test).

As the same protocol for purification was used for the WT, its variants and ΔH1–MarA, these results suggest that ΔH1–MarA shows more affinity towards non‐specific DNA than the WT MarA and the I13C/M47C and I16C/M47C variants. In order to test if it had less ability to differentiate the functional marboxes in vivo, the same reporter system as explained previously in this work was used. In this case, the IPTG‐induction and overexpression of the pET‐15b plasmid harboring ΔH1–MarA did not show any increase in the fluorescent signal, indicating that ΔH1–MarA is not able to bind and induce the transcription from the *acrAB* promoter (Figure 6a).

The accumulation assay using the dye ethidium bromide showed that ΔH1–MarA was not active in the induction of *acrAB*–*TolC*, since it was not able to reduce the accumulation of ethidium bromide when IPTG‐induced (Figure 6b). This fitted previous results, suggesting that ΔH1–MarA cannot induce the expression of the efflux pumps, maybe due to its difficulties in discerning the functional marboxes among the more than 10,000 non‐functional marboxes disseminated in the *E. coli* genome (Martin et al. 1999; Martin et al. 2002). Previously, it was shown that the overexpression of SoxS and MarA is toxic for the cell, with this toxicity being related to their accumulation and binding to non‐functional DNA sequences located upstream of genes involved in cell growth (Griffith et al. 2004). Further research is needed to determine if ΔH1–MarA could be toxic for the cell.

### 2.7 The mechanism of inhibition involving the N‐terminal helix seems to be general for the members of the AraC/XylS transcription factor family

The overlapping *marA*/*soxS*/*rob* regulon of *E. coli* contains promoters under MarA, SoxS, and Rob control (Martin and Rosner 2011). These three regulators belong to the AraC/XylS family of transcription factors characterized by the presence of a segment of ~100 amino acids that contains two HTH motifs separated by an α‐helix (Cortés‐Avalos et al. 2021; Gallegos et al. 1997). The HTH motifs are present in the DNA binding domain (DBD) and contact the DNA. The structure of these regulators showed that the DBD domain is formed by seven α‐helices, with helices 2–3 and 5–6 located in the HTH1 and HTH2, respectively, with helix 4 being a long linker, and helices 1 and 7 flanking the domain (Kwon et al. 2000; Lowden et al. 2010; Rhee et al. 1998). Some of the members of the family are composed exclusively of the DBD domain (e.g., MarA, RamA, SoxS), but others contain extra accessory domains, called companion domain (CD), that can be placed in the N‐terminus, C‐terminus, or in the middle of the protein (e.g., Rob or the methyl‐repairing protein Ada in *E. coli*) (Kwon et al. 2000; Sedgwick et al. 1988).

Due to the conservation of the sequence and 3D structure in AraC/XylS family members composed exclusively of the DBD (Figure S18), we hypothesize that the mechanism of inhibition observed in MarA could also occur in other important regulatory proteins such as RamA, SoxS, or TetR. To determine if the MarA mechanism of inhibition involving the N‐terminal helix could be a general inhibition mechanism for the whole AraC/XylS family including the members composed of the DBD and one or various accessory domains, new molecular dynamics simulations were performed. In these simulations, the cysteines introduced in I16C/M47C MarA were placed in the equivalent residues of Rob, and the dynamical behavior of the protein was compared to the one observed in MarA.

Rob is a 289 amino acid AraC/XylS family member composed of a N‐terminal DBD and C‐terminal companion domain (Kwon et al. 2000; Skarstad et al. 1993). The DBD has 51% identity and 71% similarity with MarA (Cortés‐Avalos et al. 2021; Martin and Rosner 2001). Unlike MarA and SoxS, Rob is expressed constitutively being inactive by sequestration in intracellular foci (Azam et al. 2000; Griffith et al. 2009). The crystal structures of Rob showed a different mode of DNA binding in which the DNA is unbent since only helix 3 of Rob interacts with the DNA (Kwon et al. 2000). However, molecular dynamics simulations showed that helix 6 of Rob is conformationally dynamic, and is able to move in and out of the major groove of the DNA B‐box (Corbella et al. 2021). Recently, two cryo‐EM structures have aided the understanding of the detailed molecular mechanism of Rob transcription activation (Shi et al. 2022).

Since the Rob crystal structure is only bound to the DNA through helix 3, and the disulfide bridge is thought to block the dynamics of the N‐terminal side containing this helix, we performed two sets of simulations of Rob containing the equivalent cysteines introduced in I16C/M47C MarA, namely L10C/M41C. One set of these simulations was initiated from the Rob crystal structure (PDB ID: 1D5Y) (Burley et al. 2021; Kwon et al. 2000) with the DNA unbent and only helix 3 interacting with the DNA, and a second set was initiated with the DNA bent as in the MarA crystal structures (PDB IDs: 1BL0 and 1XS9) (Burley et al. 2021; Dangi et al. 2004; Rhee et al. 1998), allowing Rob to interact with both A‐box and B‐box of the DNA (Figure S14b,c). We note that simulations of wild‐type Rob–*mar* complex in this conformation were previously performed to understand the dynamical behavior of Rob–*mar* binding recognition (Corbella et al. 2021). Based on the data from our MarA simulations, we started directly by running GaMD on both sets of complexes. We observed no marbox unbinding events in our GaMD simulations of the Rob(L10C/M41C)–*mar* complex starting from the crystal structure binding mode in any of the five replicates during 800 ns of simulation time (Figure S19a). This was not surprising since when starting simulations from a complex in which the N‐terminal HTH motif is singly bound to the DNA, the disulfide bridge was unlikely to alter the dynamics of Rob binding to the marbox. In contrast, in simulations of the Rob(L10C/M41C)–*mar* complex starting from initially bent DNA, one out of five replicates lost interactions with the A‐box of the *mar* promoter at the N‐terminal side where the disulfide bridge is inserted, while the B‐box stayed bound (Figure S19b).

**FIGURE 7:**
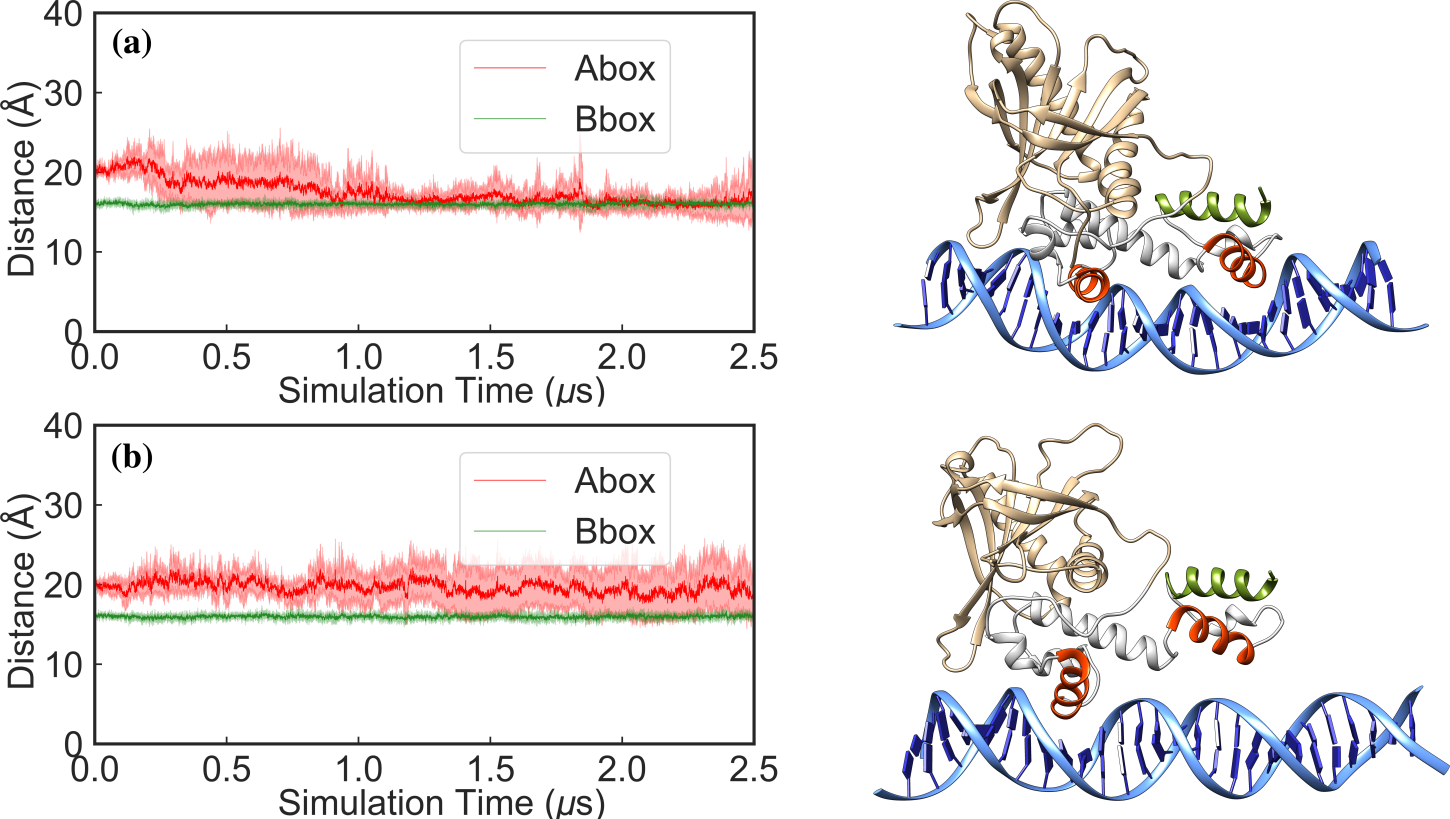
Time evolution of the distances between the helices inserted inside the major groove and the base pairs at the A‐box and B‐box (shown in red and green, respectively) during 2.5 μs of conventional MD simulations of the (a) wild‐type Rob–*mar* and (b) Rob(L10C/M41C)–*mar* complexes. Note that the starting point for these simulations was the A‐box unbound Rob(L10C/M41C)–*mar* complex obtained from our GaMD, where in (a) the double variant was reverted to wild type. Shown here also are examples of different binding conformations during our selected simulations based on visual examination of the trajectories.

To further characterize the unbinding events observed in our GaMD simulations of the Rob(L10C/M41C)–*mar* complex, we used this partially unbound structure as a starting point for new cMD simulations (5 × 2.5 μs) of both the Rob(L10C/M41C)–*mar* complex, and a system where the double cysteine variant was reverted to wild type (Figure S8h,i). We monitored the time evolution of the insertion of both Rob HTH motifs inside the A‐box and B‐box of the *mar* promoter, tracking the distances between the helices inserted inside the major groove and the base pairs at the corresponding boxes, as we did with MarA and in our previous work (Corbella et al. 2021) (i.e., between Lys35–Thr46 and nucleotides 21–22 and 39–40 for the A‐box and Gln85–Phe96 to nucleotides 12–13 and 49–50 for the B‐box) (Figure 7). We observe that the Rob(L10C/M41C) is not able to insert the helix 3 inside the A‐box of the *mar* promoter again, as observed for MarA(I16C/M47C)–*mar*, whereas the wild type reverted complex does bind the A‐box of the *mar* promoter after around 1.5 μs of simulation time despite starting from an unbound conformation, and this binding interaction remains stable until the end of our simulations (2.5 μs of simulation time; Figure 7a). Although all the simulations suggest that Rob is inhibited by the same mechanism as MarA, more experimental work is needed to confirm this mechanism of inhibition in Rob and other AraC/XylS family members.

## 3 DISCUSSION

Several studies have suggested a key role for the N‐terminal helix of MarA in DNA binding. Some of them focused on the relevance of the N‐terminal helix sequence showing by mutagenesis that the sequence of the N‐terminal helix was important for DNA binding (Gillette et al. 2000; Griffith and Wolf Jr 2002; Shah and Wolf Jr 2006). Other authors showed the coexistence of multiple energetically similar MarA conformations in solution, postulating that residues distant from the active site could alter binding affinity and ligand specificity (Dangi et al. 2001; Koulechova et al. 2015). Finally, a role of the N‐terminal helix on contacting the DNA backbone or helping to the correct positioning of the helix 3 in the major groove was proposed (Dangi et al. 2001).

From MD simulations, it was shown that the flexibility of half of the N‐terminal HTH dominates the motions of both MarA and Rob (Corbella et al. 2021; Kwon et al. 2000). In the present work, we wondered if impeding the motions of the N‐terminal helix of MarA, that is, affecting its N‐terminal domain dynamics could prevent DNA binding. To do this, we synthesized two double cysteine MarA variants that allowed us to study the effect on the immobilization of the N‐terminal helix of MarA by the formation of disulfide bonds. After disulfide bond formation, the I16C/M47C variant adopted an inhibited conformation, suggesting that the design of inhibitors based on targeting MarA N‐terminal domain dynamics can be a good strategy to prevent DNA binding and the consequent overexpression of AcrAB–TolC.

By MD simulations, we show that this inhibited conformation also avoids the binding of the two domain transcriptional factor Rob to the DNA. Members of the AraC/XylS family, including MarA or Rob, regulate relevant functions such as biofilm formation, quorum sensing, pathogenicity and motility. Targeting these proteins by inhibitors can contribute to the development of new treatments against multidrug‐resistant bacteria.

To quantify the biological significance of the inhibited MarA conformation, we measured the efflux of AcrAB–TolC in cells that allow the formation of disulfide bonds in the cytoplasm. However, the redundancy between the three closely related regulators of *acrAB*–*tolC* (MarA, SoxS, and Rob), made detection of phenotypic differences difficult. To observe differences would potentially require a triple knockout (∆*marA*, ∆*soxS*, ∆*rob*) in the Shuffle T7 express cells, but the already highly modified genetic background of these cells would complicate the analysis of any obtained result. Even with this limitation of our model, the ability of the I16C/M47C variant to bind marboxes only when the disulfide bond is broken was evidenced by the EMSAs, molecular dynamic simulations and the different results obtained with the reporter systems in the T7 express and shuffle T7 express background.

The inhibited conformation described in this work seems to be shared by both, the AraC/XylS family members composed exclusively of the DBD, such as MarA, and the members having extra accessory domains, such as Rob. The identification of this new structural element, distant to the helices involved in making direct contacts with the DNA, opens the possibility for the design of new inhibitors targeting the dynamics of the N‐terminal helix of the AraC/XylS transcription factors. The high degree of conservation of the N‐terminal helix of the three closely related regulators suggests the possibility of designing a common inhibitor that impedes the dynamics of the N‐terminal helix of the three regulators to prevent their DNA binding.

## 4 MATERIALS AND METHODS

### 4.1 Bacterial strains and growth conditions

The strains used in this work were T7 express and shuffle T7 express *E. coli* cells (New England Biolabs). All strains were grown in Luria‐Bertani (LB) broth at 37°C at 200 rpm. Antibiotics were purchased from Alfa Aesar (Ward Hill, MA) and Merck (Darmstadt, Germany).

### 4.2 Cloning and site‐directed mutagenesis

The plasmid p*marA* was generated by PCR‐amplification of the *marA* gene from *E. coli* MG1655 genomic DNA cloned into the *Nde*I and *HindIII* sites of pET‐15b. This cloning involved the addition of a 6xHis‐tag to the N‐terminus of the translated protein. All site‐directed mutagenesis (SDM) reactions were carried out using the Quick‐Change Lightning SDM Kit (Agilent), the primers listed in Table S1, and the plasmid p*marA* as template. The double cysteine variants were prepared in two steps, by introducing the cysteines in series. The mutations were verified by sequencing (Eurofins Genomics, UK). The N‐terminal deletion of MarA (called ΔH1–MarA) was obtained by amplification of the nucleotides corresponding to amino acids Glu25 to Ser129 by using the MarA_NdeI_deletionH1_F and MarA_HindIII_R primers (Table S1). This fragment was cloned into the *Nde*I and *HindIII* sites of pET‐15b as previously described for the wild‐type (WT) *marA*.

### 4.3 Protein purification

WT MarA, its double cysteine variants I13C/M47C and I16C/M47C, and ΔH1–MarA were expressed in T7 express *E. coli* cells (New England Biolabs) grown to OD600≈0.5 and induced with 0.5 mM IPTG for 3 h at 37°C. The cells were harvested by centrifugation (5000*g*, 15 min, at 4°C), resuspended in 50 mM Tris–HCl pH 8, 1M NaCl, 0.03 mg/mL DNase I (Sigma), supplemented with a protease inhibitor cocktail pill (Roche), and lysed by sonication. Inclusion bodies containing MarA were collected by centrifugation at 75,000*g* for 30 min and washed with 50 mM Tris–HCl pH 8, 1M NaCl, 2M urea and centrifuged at 75,000*g* for 30 min. Inclusion bodies were solubilized with 50 mM Tris–HCl pH 8, 1M NaCl, 7M urea and isolated by high‐speed centrifugation (75,000*g*, 30 min). The supernatant was loaded in a 1 mL HisTrap (GE Healthcare), equilibrated with 50 mM Tris–HCl pH 8, 1M NaCl, 7M urea, 50 mM imidazole and eluted by increasing the concentration of imidazole up to 300 mM. Purified MarA was buffer exchanged into 50 mM Hepes pH 8, 1M NaCl by dialysis. For the cleavage of the His‐tag, thrombin sepharose beads (BioVision) were incubated with the protein overnight at room temperature. The cleaved MarA was separated by using a second round of affinity chromatography. The protein was stored at −80°C after adding 20% glycerol. As ΔH1–MarA was recovered in a complex with DNA after purification, some variations were added to the protocol such as the increase in DNase (0.06 mg/mL) and the addition of 2.5 mM MgCl_2_, 0.2 mM CaCl_2_ to improve the conditions for DNase I activity. These modifications did not allow the recovery of the pure unbound ΔH1–MarA protein.

### 4.4 Electrophoretic mobility shift assay

For electrophoretic mobility shift assay (EMSA) experiments, the 200‐bp DNA fragments containing the *marRAB* or *acrAB* marboxes were prepared by PCR using the oligonucleotides listed in Table S1 and *E. coli* MG1655 genomic DNA as template. The 30‐bp complementary DNA fragments corresponding to the *marRAB* or *acrAB* marboxes with five nucleotides in each flank were purchased from Eurogentec (Seraing, Belgium; Table S1). The DNA fragments (15 and 200 nM when using 200‐bp and 30‐bp DNA fragments, respectively) were incubated with MarA in a buffer containing 20 mM Hepes pH 8, 100 mM MgCl_2_, 100 mM EDTA, 0.3 mg/mL BSA, and dithiothreitol (DTT) when indicated. 0.8 μM of purified *E. coli* MarA WT or its variants were added and the mix was incubated for 20 min at 37°C (final volume 10 μL)/jbc.M. The samples were mixed with 6X EMSA gel‐loading solution (component D, Electrophoretic Mobility Shift Assay (EMSA) Kit, Invitrogen) and loaded onto an 8% native acrylamide gel and visualized with SYBR green (Invitrogen).

### 4.5 Reporter system

A reporter system built in a pACYC177 plasmid and composed of the *E. coli acrAB* promoter followed by the GFP protein (named pACYC177‐RS) was co‐transformed with the pET‐15b empty vector, or *pmarA* harboring the WT, ΔH1–MarA, I13C/M47C, or I16C/M47C *marA* genes into T7 express or shuffle T7 express *E. coli* cells. Shuffle T7 express *E. coli* cells lack important reductases located in the *E. coli* cytoplasm generating an oxidative folding that allows the formation of disulfide bonds in the cytoplasm. We chose pACYC177 since the origin of replication is compatible with pET‐15b, plasmid in which WT MarA and the variants were cloned. Shuffle T7 express cells were grown up to OD_600_ = 0.5 and induced with 0.5 mM IPTG for 90 min at 37°C while T7 express cells were grown to OD_600_ = 0.5 and induced with 0.5 mM IPTG for 30 min at 37°C. The cells were harvested by centrifugation, the pellet was resuspended with MOPS minimal medium, and 190 μL of all the strains were placed in a 96‐well black/clear bottom plate (Thermo Fisher) and the OD_600_ and fluorescence was measured. The fluorescence signals were normalized using the number of cells for every culture. Data presented are the mean of two biological replicates.

### 4.6 Western blotting

Wild type and variants of MarA were expressed in the shuffle T7 express *E. coli* strain. Cultures were grown to an OD_600_ of 0.5 and induced with 0.5 mM IPTG for 90 min at 37°C. Cells were harvested and lysed in 50 mM phosphate buffer pH 8, 1M NaCl, supplemented with complete EDTA‐Free Protease Inhibitor tablets (Roche, Switzerland) using sonication. Membrane fractions were harvested, separated using an any kD precast SDS‐PAGE gel (Biorad, UK), and transferred to a nitrocellulose membrane by using the iBlot Dry blotting system (Thermofisher Scientific). The membrane was rinsed in phosphate‐buffered saline solution with 0.1% Tween‐20 (PBST) and blocked with PBST including 5% (w/v) milk powder for 60 min at room temperature. The membrane was then incubated overnight at 4°C with a 1:1000 concentration of anti‐6xHis tagged monoclonal antibody produced in mouse (ABD Serotec Biorad, UK). Then, the membrane was washed with PBST (three times for 10 min) and incubated with a 1:10000 concentration of secondary Licor IRDye 680RD‐goat‐anti‐mouse‐IgG (in TBST with 5% (w/v) milk powder for 60 min, darkness) and washed with TBS (three times for 10 min). Fluorescence detection was performed using the LiCor Odyssey (Model 9120) 3.0 imaging system.

### 4.7 Ethidium bromide accumulation assay

T7 express *E. coli* cells were transformed with the pET‐15b empty vector, or *pmarA* harboring the WT or ΔH1–MarA *marA* genes. The cells were grown up to OD_600_ = 0.4 and induced by 0.5 mM IPTG for 15 min at 37°C. Later, the cells were harvested by centrifugation and the pellet was resuspended with potassium phosphate buffer pH 7, containing 1 mM MgCl_2_. After adjusting the OD_600_ to 0.2, 200 μL of each culture was inoculated into the wells of a black polystyrene microtiter tray (Greiner CELLSTAR 96 well plates flat bottom) and a 10 μL injection of 50 μg/mL ethidium bromide (EtBr) was applied to each well. The fluorescence signal was measured over 66 min at excitation and emission wavelengths of 530 and 600 nm, respectively, in a FLUOstar Optima. Data presented are the mean of three biological replicates.

### 4.8 Mass spectrometry

The molecular mass of WT MarA, I13C/M47C and I16C/M47C variants, with and without a previous step of reduction with 1 mM dithiothreitol (DTT), was determined using a UHPLC‐MS Nexera‐i LC‐2040C instrument (Shimadzu, Kyoto, Japan) with a Kinetex PS‐C18 column (50 × 2.1 mm, 2.6 μm; Phenomenex). The samples were eluted with a linear gradient starting at 5% of solvent B (0.08% formic acid in MeCN) and reaching 95% of solvent B over 3 min at a 0.6 mL/min flow rate, with UV detection at 220 nm. The theoretical monoisotopic masses were determined by the GPMAW software (version 12.2). The calculated and observed monoisotopic masses of each protein are shown in Tables S2–S6. ESI‐MS spectra for WT MarA, I13C/M47C, and I16C/M47C in the absence (oxidized conditions) and presence (reduced conditions) of 1 mM DTT are included in Data S1.

### 4.9 Intrinsic fluorescence measurements

Tryptophan fluorescence measurements were performed on a Spark multimode microplate reader (Tecan, Switzerland). The excitation wavelength was fixed at 280 nm, and the emission spectra were recorded at 25°C from 300 to 500 in a 96‐well black bottom microplate (Greiner). A bandwidth of 7.5 nm was used for the excitation and emission beams, number of flashes was 30, integration time 40 μs, and gain 150. WT MarA and its variants spectra were measured at 1.2 μM in 50 mM Hepes buffer, pH 8, 1M NaCl at room temperature. The normalized fluorescence values represent the average of three replicates that were previously corrected by subtracting the baseline corresponding to the buffer without protein.

### 4.10 Bioinformatic tools

The protein sequences shown in this work were obtained by using the Uniprot Database (https://www.uniprot.org/) and the alignments were done by using Clustal Omega in the EBI (European Bioinformatics Institute) webserver (http://www.ebi.ac.uk/clustalw/) (The UniProt Consortium 2023; Goujon et al. 2010). The PDBs were obtained from the Protein Data Bank (https://www.rcsb.org/) (Berman et al. 2000; Berman et al. 2003). The figures and structural superpositions were obtained by using Chimera (Pettersen et al. 2004).

### 4.11 System preparation for conventional and Gaussian accelerated molecular dynamics simulations

Starting coordinates for all the simulations in this work were taken from the crystal structure of MarA in complex with the *mar* promoter available in the Protein Data Bank (PDB ID: 1BL0) (Berman et al. 2000; Rhee et al. 1998). Two sets of simulations were performed, one set describing free unbound MarA, and a second describing the MarA–*mar* complex, in both MarA's wild‐type form, as well as the MarA(I13C/M47C) and MarA(I16C/M47C) variants. Starting structures for simulations of these variants were created by substituting the corresponding residues to cysteine using the Dunbrack 2010 Rotamer Library (Shapovalov and Dunbrack Jr 2011), as implemented in UCSF Chimera, v. 1.14 (Pettersen et al. 2004). In each case, rotamers that facilitated the formation of a disulfide bridge were selected as simulation starting points, where the disulfide bridge was imposed and thus formed at the first equilibration steps. In the case of simulations of the MarA–DNA complex, the DNA sequence in the crystal structure was slightly modified and extended at the two ends to match the 30‐bp fragment used experimentally (5′GAACCGATTTAGCAAAACGTGGCATCGGTC3′). Simulations of free MarA were initiated by manually deleting the DNA promoter from the starting structure for simulations of the analogous complexes.

Finally, in addition to the initial set of simulations starting from the DNA‐bound structures of the MarA(I13C/M47C)–*mar* and MarA(I16C/M47C)–*mar* complexes (based on the wild‐type MarA–*mar* crystal structure), we also performed a second set of “DNA‐unbound” simulations of wild‐type MarA (i.e., a C16I/C47M reversion), MarA(I16C/M47C), and MarA(I16C/M47C) with a broken disulfide bridge (named I16C/M47C^broken^), where the starting structure for each set of simulations was extracted from Gaussian accelerated molecular dynamics (GaMD) simulations of the MarA(I16C/M47C)–*mar* complex (Miao et al. 2015). In the latter case, we selected as our starting point a representative snapshot from the GaMD simulations where the N‐terminal HTH motif of MarA has lost its interaction with the A‐box of the *mar* promoter. Conventional molecular dynamics (cMD) simulations were then performed on the MarA(I16C/M47C)–*mar* complex extracted from the GaMD simulations, as well as on (1) the corresponding structure of the wild‐type MarA–*mar* complex, where both mutations were reverted back to wild type, (2) the corresponding complex where the disulfide bridge was broken by addition of a hydrogen atom to both C16 and C47, and (3) the corresponding complex where the two cysteine residues forming the disulfide bridge were mutated to serine (I16S/M47S). The reversion to wild‐type MarA was again performed using the Dunbrack 2010 Rotamer Library as implemented in UCSF Chimera (Pettersen et al. 2004; Shapovalov and Dunbrack Jr 2011), and the relevant rotamers selected were chosen to mimic those in the crystallographic structure of wild‐type MarA. Relevant data concerning the molecular dynamics simulations are available for download from Zenodo, https://doi.org/10.5281/zenodo.7404276.

### 4.12 Conventional molecular dynamics simulations

Conventional molecular dynamics (cMD) simulations were performed following the same protocol as our prior study of MarA in complex with various DNA promoter sequences (Corbella et al. 2021). In brief, all simulations were performed using the Amber ff14SB force field (Maier et al. 2015) to describe MarA and Rob, the Parmbsc1 force field to describe the DNA (Ivani et al. 2016), and the CUDA version of the PMEMD module (Götz et al. 2012) of the AMBER 18 simulation package (Case et al. 2018). Five independent production runs of 2.5 μs of length each with different initial velocities were performed for each system, as summarized in Table S7. Further simulation details can be found in Corbella et al. (2021).

### 4.13 Gaussian accelerated molecular dynamics simulations

Gaussian accelerated MD (GaMD) (Miao et al. 2015) is an enhanced sampling method that works by adding a non‐negative boost potential, that follows a Gaussian distribution, to smoothen the potential energy surface (PES) of the system, thus decreasing the energy barriers between minima and accelerating transitions between low‐energy states. In this work, GaMD was applied to simulations of both the MarA(I13C/M47C)–*mar* and MarA(I16C/M47C)–*mar*, and the Rob(L10C/M41C)–*mar* complexes after an initial 700 ns of conventional molecular dynamics simulations of the DNA‐bound complexes had completed. A *dual‐boost* scheme was applied for the boost potential, which considers the acceleration potentials to both dihedrals and the total potential energy of the system simultaneously. The system threshold energy was set to *E* = *V*_max_ and a timestep of 4 fs was used, following from our previous conventional MD setup. The 4 fs time step was achieved via the hydrogen mass repartition scheme (Hopkins et al. 2015) (which involves altering the mass of hydrogen atoms to 3.024 amu to allow for the larger time step), using the PARMED module of AMBER 18 (Case et al. 2018). After each cMD simulation, 84 ns of GaMD equilibration was performed in the NVT ensemble, during which time the boost potential was updated every 7.2 ns, allowing equilibrium values of the acceleration parameters *E* and *k*_0_ to be reached (for details about what these parameters mean, see Miao et al. (2015)). Finally, 720 ns of production GaMD simulations were performed, considering five independent replicas for each system, comprised of 700 ns of pre‐equilibration cMD followed by 84 ns of GaMD equilibration and finally 720 ns of GaMD production runs per replica. This led to a cumulative total of ~8 μs of GaMD simulations.

### 4.14 Analysis of molecular dynamics simulations

All analysis of MD simulations was performed using CPPTRAJ (Roe and Cheatham 3rd 2013). Principal component analysis (PCA) was performed on the combined MD simulations of all free MarA systems by first root‐mean‐square (RMS) fitting to all C_α_ carbon atoms, using the crystal structure as a reference, and then performing PCA on all the C_α_ carbon atoms. The most‐populated structures were calculated by performing agglomerative hierarchical clustering on the root mean square deviation (RMSD) of C_α_‐atoms to yield five discrete clusters. The centroid of the most‐populated cluster was then selected for further characterization. The root mean square deviations (RMSD) and fluctuations (RMSF) of the C_α_‐atoms were calculated over the five replicas. Secondary structural propensities for all residues of free MarA were calculated using the DSSP method of Kabsch and Sander (Kabsch and Sander 1983).

## AUTHOR CONTRIBUTIONS

**Marina Corbella:** Conceptualization; methodology; investigation; visualization; writing – original draft; writing – review and editing. **Cátia Moreira:** Investigation; visualization; writing – review and editing. **Roberto Bello‐Madruga:** Investigation; visualization; writing – original draft; writing – review and editing. **Marc Torrent Burgas:** Supervision; writing – review and editing. **Shina C. L. Kamerlin:** Conceptualization; methodology; supervision; writing – review and editing; writing – original draft. **Jessica M. A. Blair:** Conceptualization; methodology; supervision; writing – original draft; writing – review and editing. **Enea Sancho‐Vaello:** Conceptualization; methodology; investigation; visualization; supervision; writing – review and editing; writing – original draft.

## CONFLICT OF INTEREST STATEMENT

The authors declare no conflicts of interest.

## Supporting information


**Data S1.** Supporting Information.
