# Supplementary material for: Targeting MarA N‐terminal domain dynamics to prevent DNA binding
Source: Protein Sci. 2024 Dec 11;34(1):e5258. doi: 10.1002/pro.5258 (PMC11633057; doi:10.1002/pro.5258)
Supplement: Supplementary file 1 — Data S1. Supporting Information. [file PRO-34-e5258-s001.docx]

**Supporting Information for**

Targeting MarA N-terminal domain dynamics to prevent DNA binding

Marina Corbella^1,2,†^, Cátia Moreira^1^, Roberto Bello-Madruga^3^, Marc Torrent Burgas^3^, Shina C.L. Kamerlin^1,4^, Jessica M.A. Blair^5^, Enea Sancho-Vaello^3,5,††,*^

^1^ Science for Life Laboratory, Department of Chemistry – BMC, Uppsala University; Uppsala, S-751 23, Sweden.

^2^ Departament de Quı́mica Inorgànica i Orgànica (Secció de Quı́mica Orgànica) & Institut de Quı́mica Teòrica i Computacional (IQTCUB), Universitat de Barcelona, Martı́ i Franquès 1; 08028 Barcelona, Spain.

^3^ Department of Biochemistry and Molecular Biology, Universitat Autònoma de Barcelona; 08193 Cerdanyola del Vallès, Spain

^4^ School of Chemistry and Biochemistry, Georgia Institute of Technology, 901 Atlantic Drive NW; Atlanta, GA 30332-0400.

^5^ College of Medicine and Health, Department of Microbes, Infection and Microbiomes, Institute of Microbiology and Infection, University of Birmingham; Edgbaston, Birmingham, B15 2TT, United Kingdom.

** Correspondence: Enea Sancho-Vaello, Department of Biochemistry and Molecular Biology, Biosciences Faculty, Building C, Office C2/427, Campus de la UAB · 08193 Bellaterra, Phone: + 34 935 814 889, Email:* [*enea.sancho@uab.cat*](mailto:enea.sancho@uab.cat)

Present addresses:

† Marina Corbella, Departament de Quı́mica Inorgànica i Orgànica (Secció de Quı́mica Orgànica) & Institut de Quı́mica Teòrica i Computacional (IQTCUB), Universitat de Barcelona, Martı́ i Franquès 1; 08028 Barcelona, Spain.

†† Enea Sancho-Vaello, Department of Biochemistry and Molecular Biology, Universitat Autònoma de Barcelona, Cerdanyola del Vallés 08193, Spain.

**This PDF file includes:**

Figures S1 to S19 page 2

Tables S1 to S7 page 23

SI References page 32

Fig. S1.

**
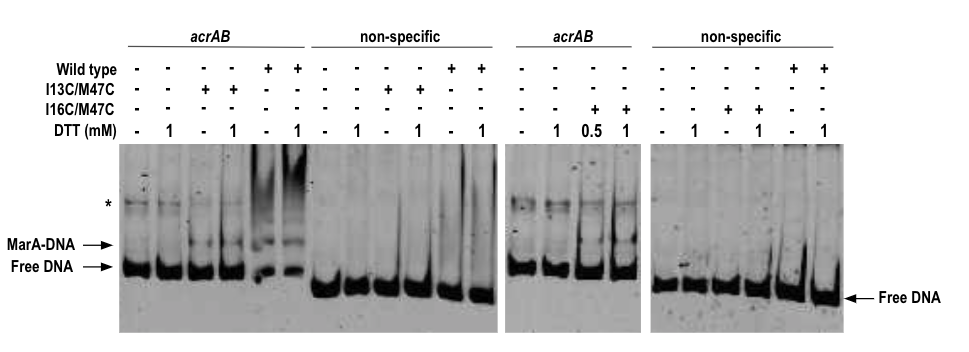
**

EMSA for WT MarA and double cysteine variants I13C/M47C and I16C/M47C by using 200-bp DNA fragments harbouring the *acrAB* marbox or a non-specific sequence. On top of the lanes corresponding to the *acrAB* fragment, there is an unexpected band linked to contamination (see the asterisk).

Fig. S2.


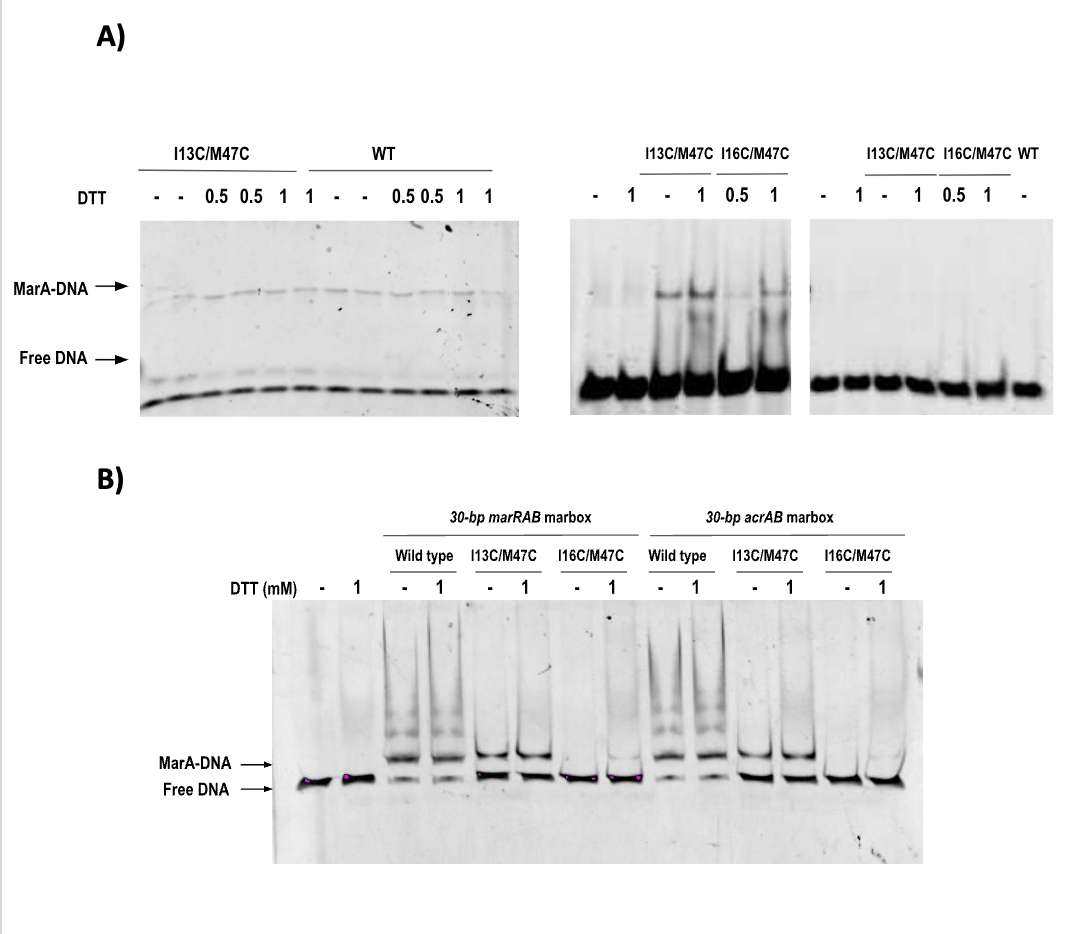


**(A)** EMSA for WT MarA and its double cysteine variants, I13C/M47C and I16C/M47C, in the presence and absence of DTT, by using 30-bp DNA fragments containing the *marRAB* marbox (left and middle panel) or a 30-bp non-specific fragment (right panel) (see sequence in Supplementary Materials Table S1). I13C/M47C and WT MarA can bind the DNA fragments in the presence and absence of DTT. I16C/M47C only can bind DNA when DTT is present (DTT concentrations were 0.5 or 1 mM). None of the three proteins were able to shift a 30-bp non-specific DNA fragment. **(B)** EMSA assay by using the 30-bp *marRAB* and *acrAB* marboxes flanked by 5 amino acids in each flank (see sequences in Supplementary Materials Table S1) and the His-tag cleaved wild type MarA and double cysteine variants I13C/M47C and I16C/M47C. The pattern of bands is similar to the one obtained when working with the His-tagged proteins (Figure 2 and Supplementary Figure S1).

**Fig. S3.**


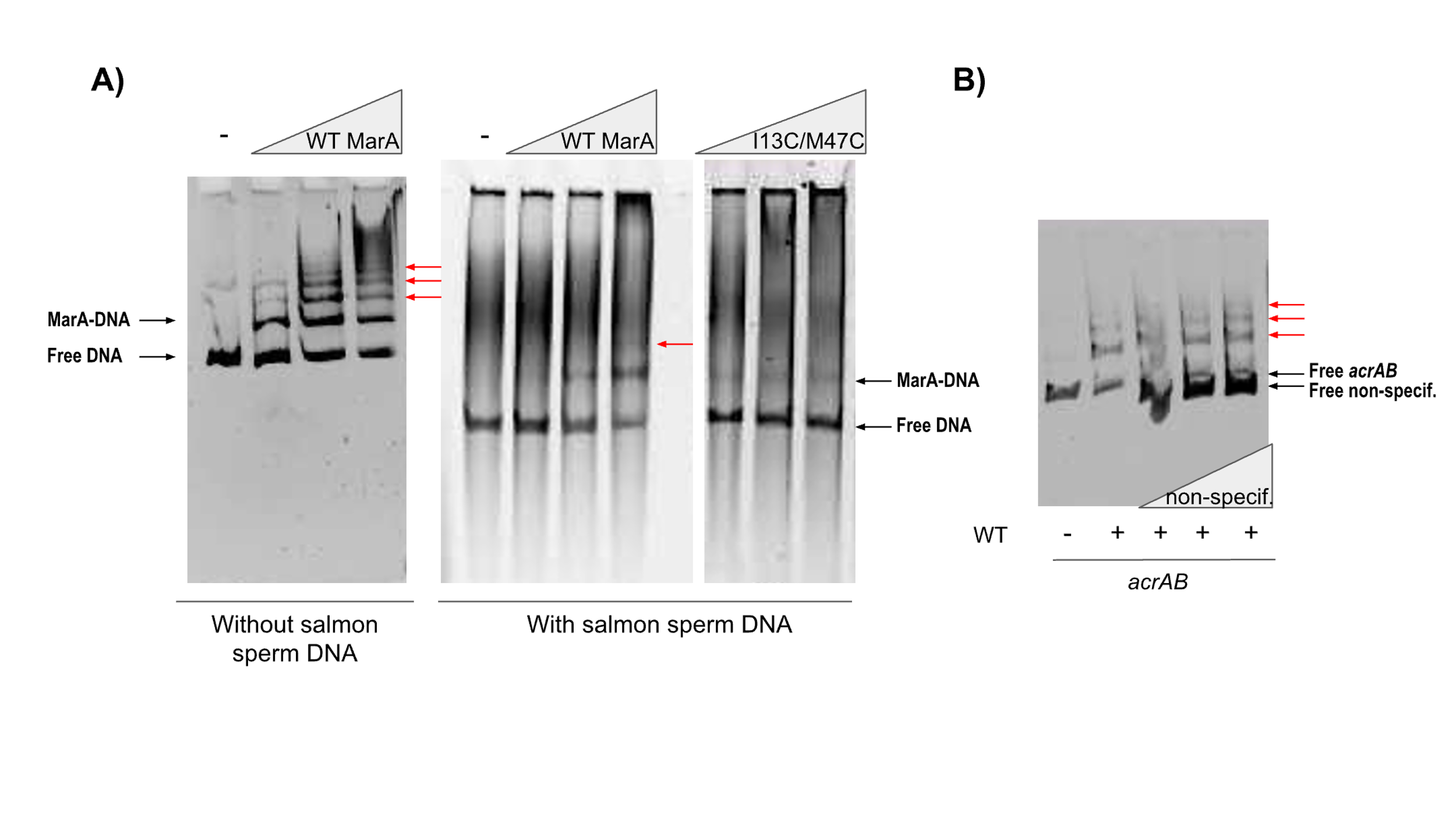


Competitive EMSAs. **(A)** EMSA for WT MarA and I13C/M47C variant (0.8 μM) in the absence and presence of an excess of salmon sperm DNA (25 ng/μl) by using 200-bp DNA fragments containing the *acrAB* marbox (15 nM, or 2 ng/μl). As our method of detection (SYBR green) stains all the DNA present in the assay, the salmon sperm DNA was also stained complicating the visualisation of the shifted bands. **(B)** EMSA for WT MarA in the presence of an excess of 200-bp non-specific DNA (15 nM, 21 nM, and 30 nM). This non-specific DNA fragment was used as a competitor DNA since WT MarA and its variants were not able to shift it (Supplementary Figure S1). In both (A) and (B), the multi-band pattern exhibited by the WT remains in the presence of the non-specific competitors (red arrows). This fact indicates that the multiple bands appearing in the EMSA without salmon sperm DNA correspond to specific binding.

Fig. S4.


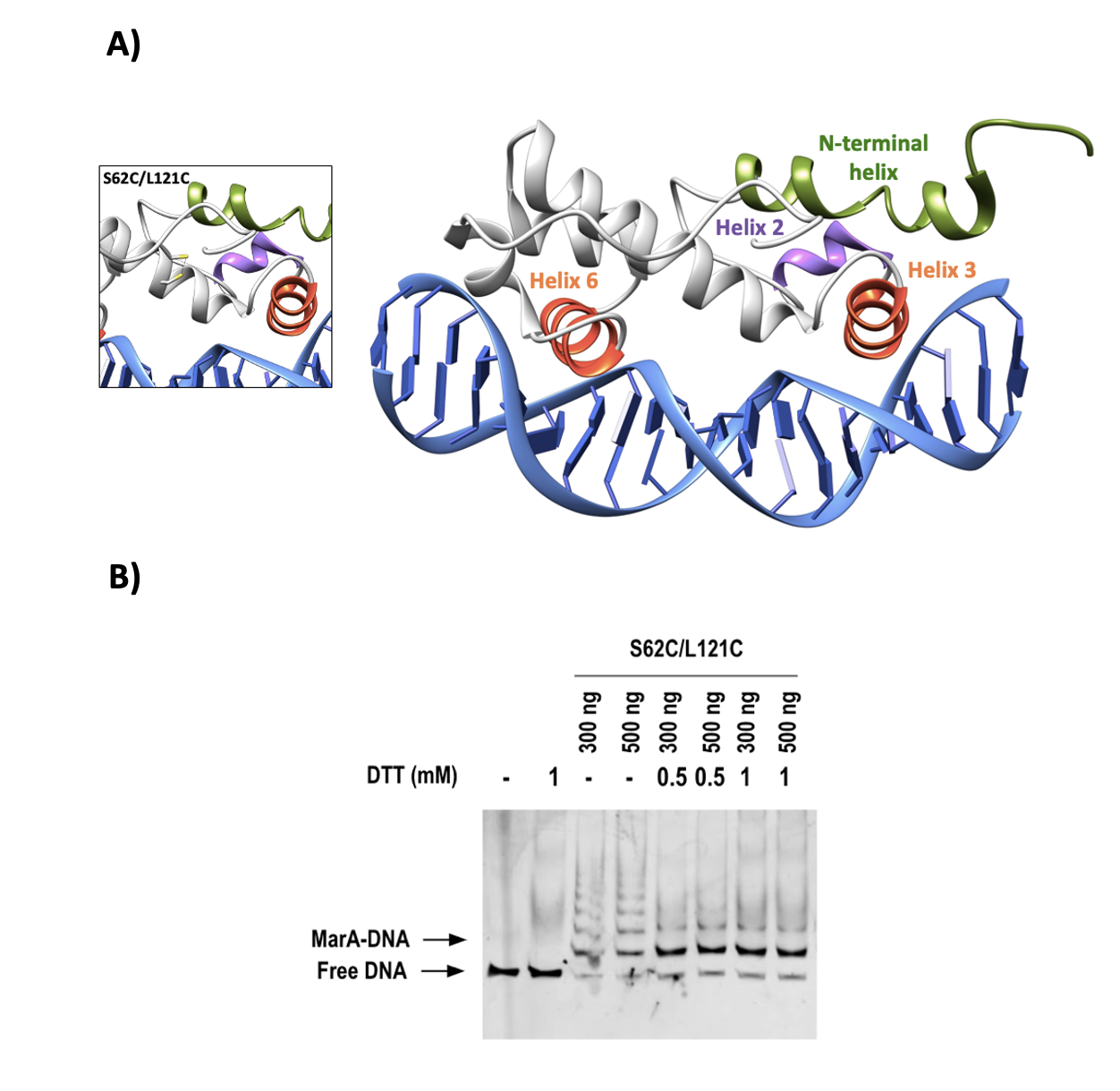


**(A)** Structure of the WT MarA (PDB 1XS9) ^29,36^ as shown in Figure 1. Close-up showing the predicted structural model for the S62C/L121C variant, in which the formation of the disulfide bond blocks the movement of the C-terminal tail since the residues 62 and 121 are located in the helix 4 and the C-terminal tail, respectively. **(B)** EMSA for the double cysteine variants S62C/L121C in the presence and absence of DTT by using 200-bp DNA fragments containing the *marRAB* marbox. The disulfide bond in the S62C/L121C variant was designed to not affect the DNA binding in either the oxidised or reduced form. The S62C/L121C variant is able to bind the marbox in the absence and presence of DTT.

Fig. S5.

A)


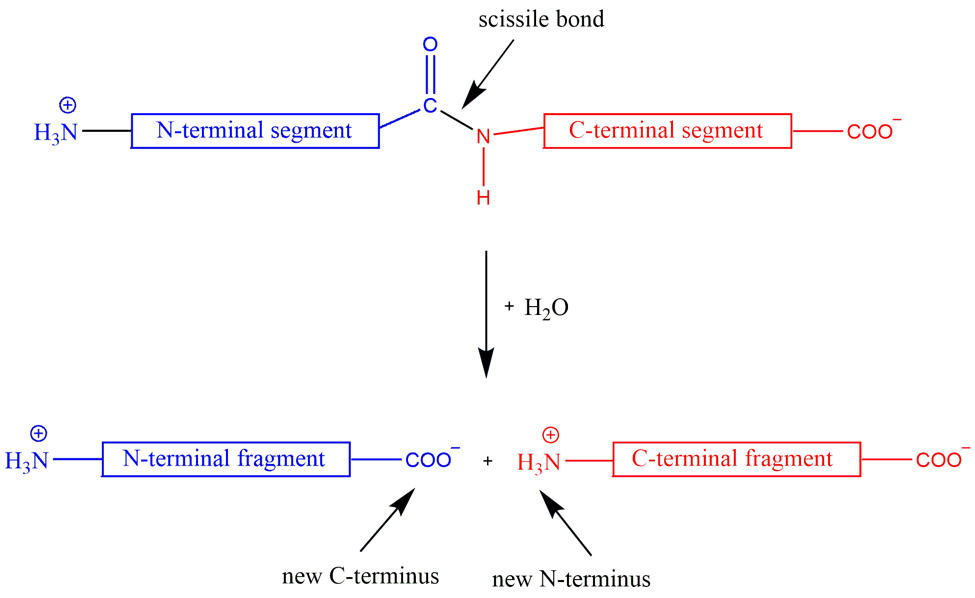


B)


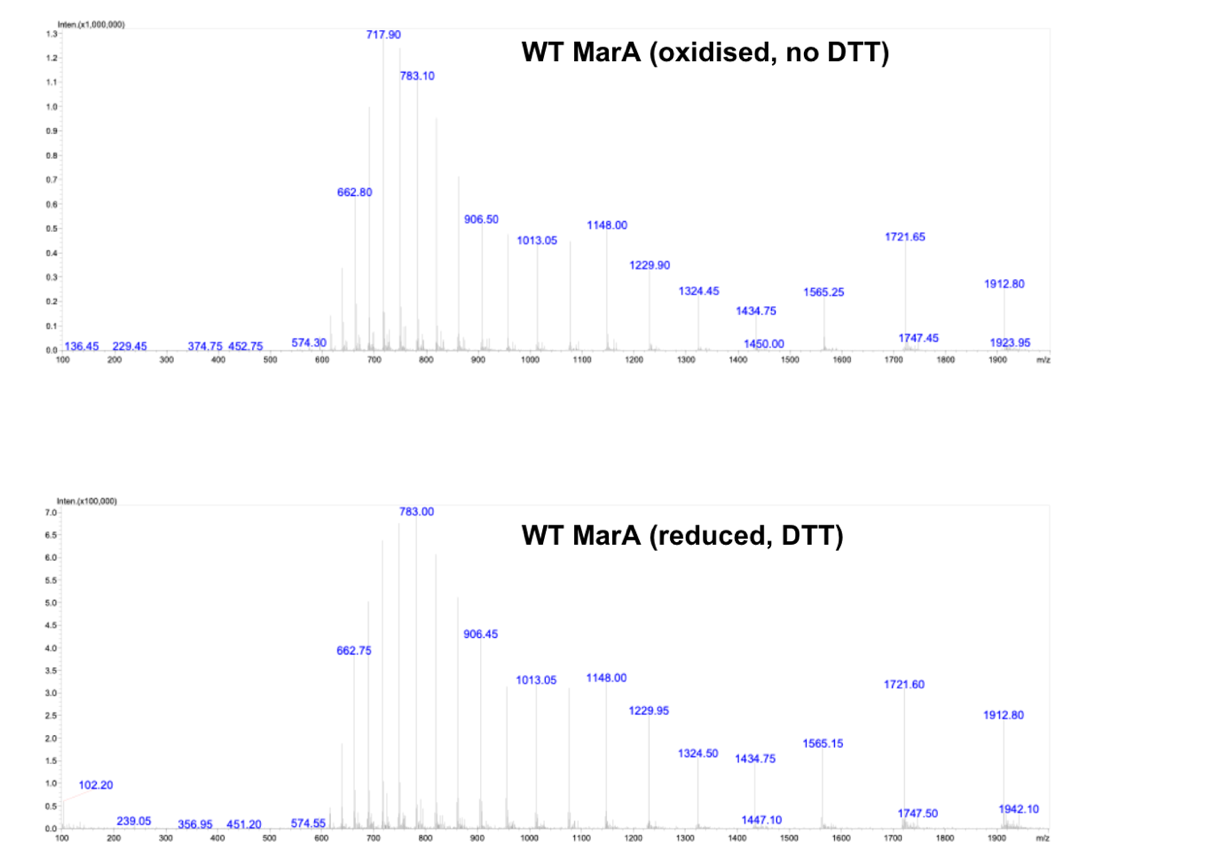


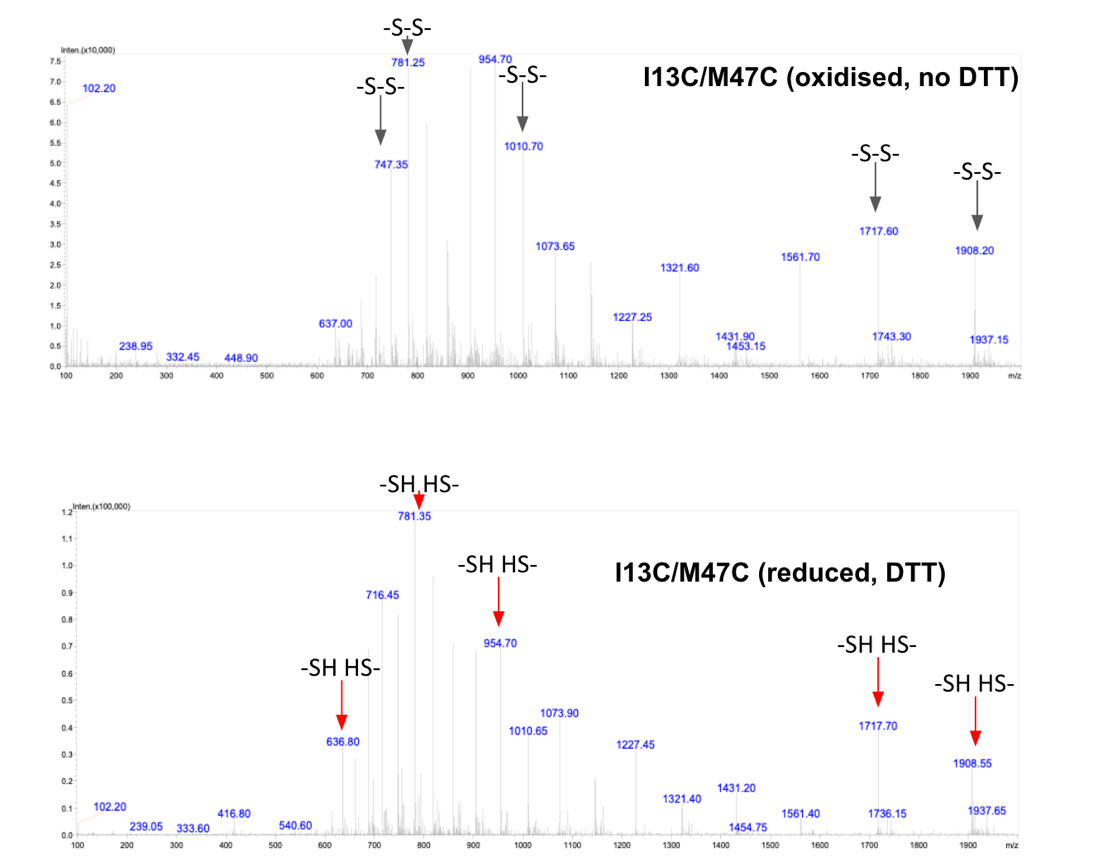


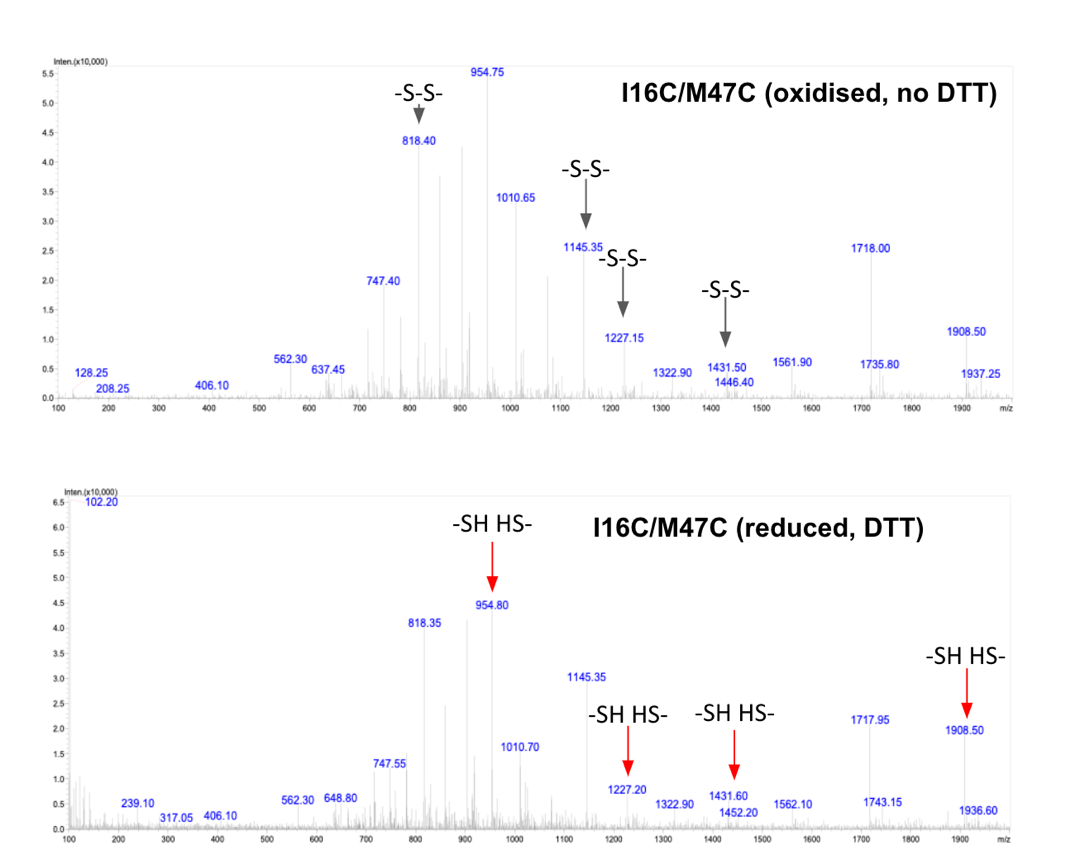


**(A)** N-terminal methionine excision, a common co-translational modification observed in WT MarA and the I13C/M47C and I16C/M47C variants. Taking the N-terminal methionine into consideration, the observed molecular masses were in excellent agreement with the theoretical one. **(B)** HPLC-ESI/MS chromatograms for WT MarA, I13C/M47C and I16C/M47C in the absence (oxidised conditions) and presence (reduced conditions) of 1 mM DTT.

Fig. S6.


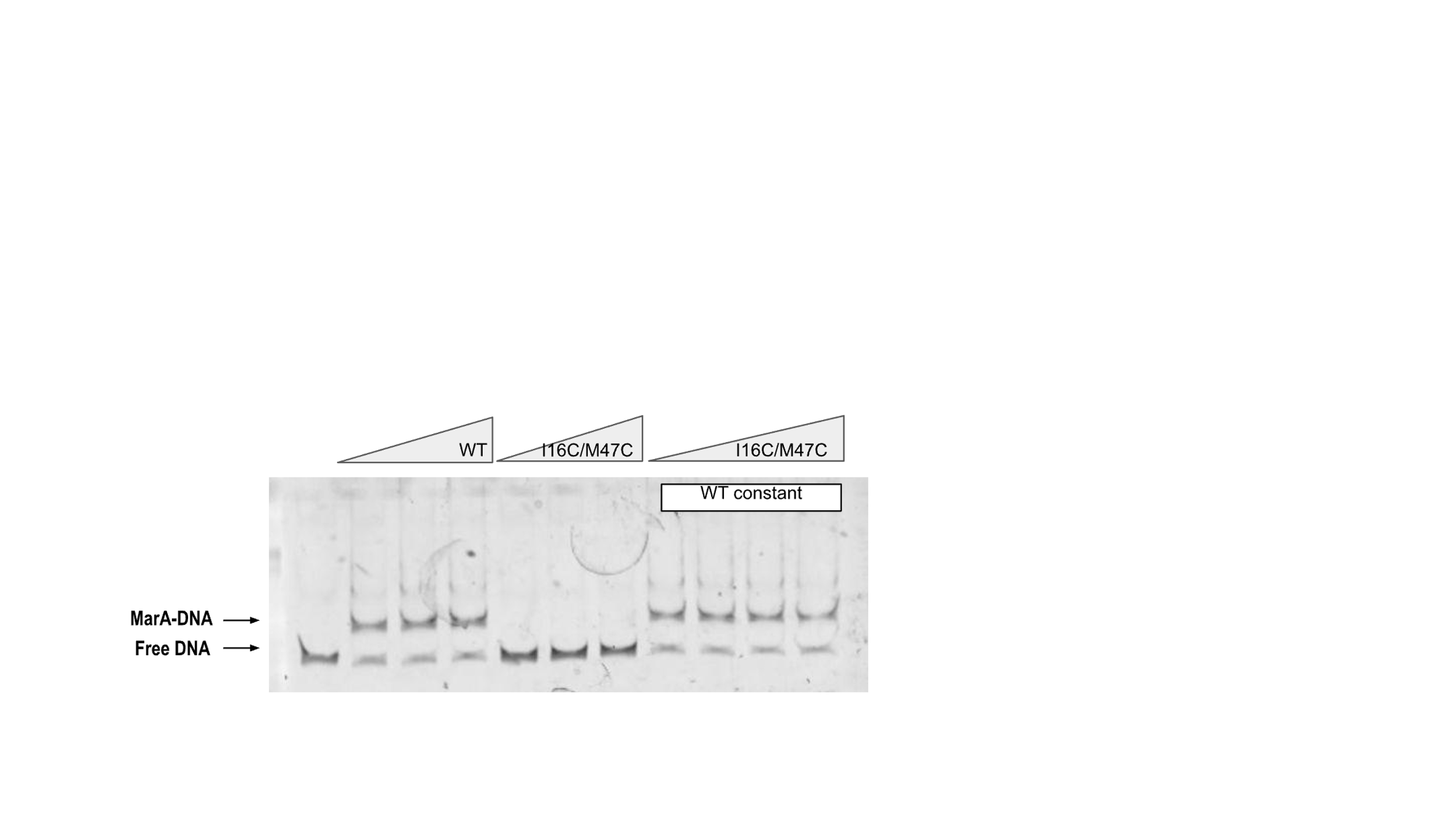


EMSA using 200 bp-DNA fragments containing the *marRAB* marbox. Wild type (WT) MarA was tested alone (range 200 – 600 ng), I16C/M47C alone (range 100 – 300 ng) and both variants together (increasing I16C/M47C concentrations (100 – 350 ng) maintaining the WT concentration constant (200 ng)). In this assay, DTT was not added. The addition of increasing concentrations of I16C/M47C does not seem to affect WT MarA binding to the marbox.

Fig. S7.


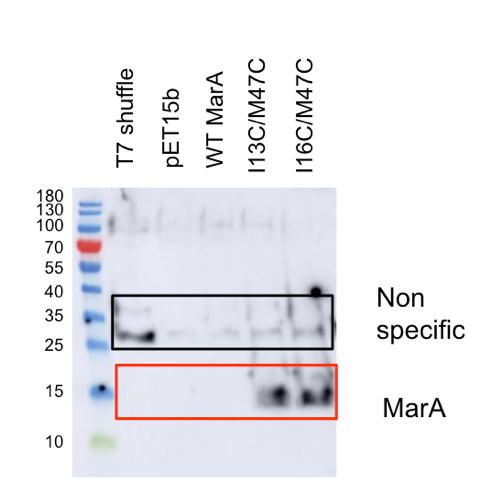


Western blot to detect the His-tagged MarA protein in shuffle T7 express cells, and shuffle T7 express cells harbouring the pET-15b empty vector, or pET-15b harbouring WT MarA, or I13C/M47C, or I16C/M47C. Western blotting was performed using an HRP-conjugated anti-His tag antibody. MarA can be detected in the cells containing the variants, but not in the shuffle T7 express cells, pET-15b empty vector or WT MarA. The non-specific bands appearing in all the tested strains indicates that this antibody was less selective than the one used in Figure 3B.

Fig. S8.


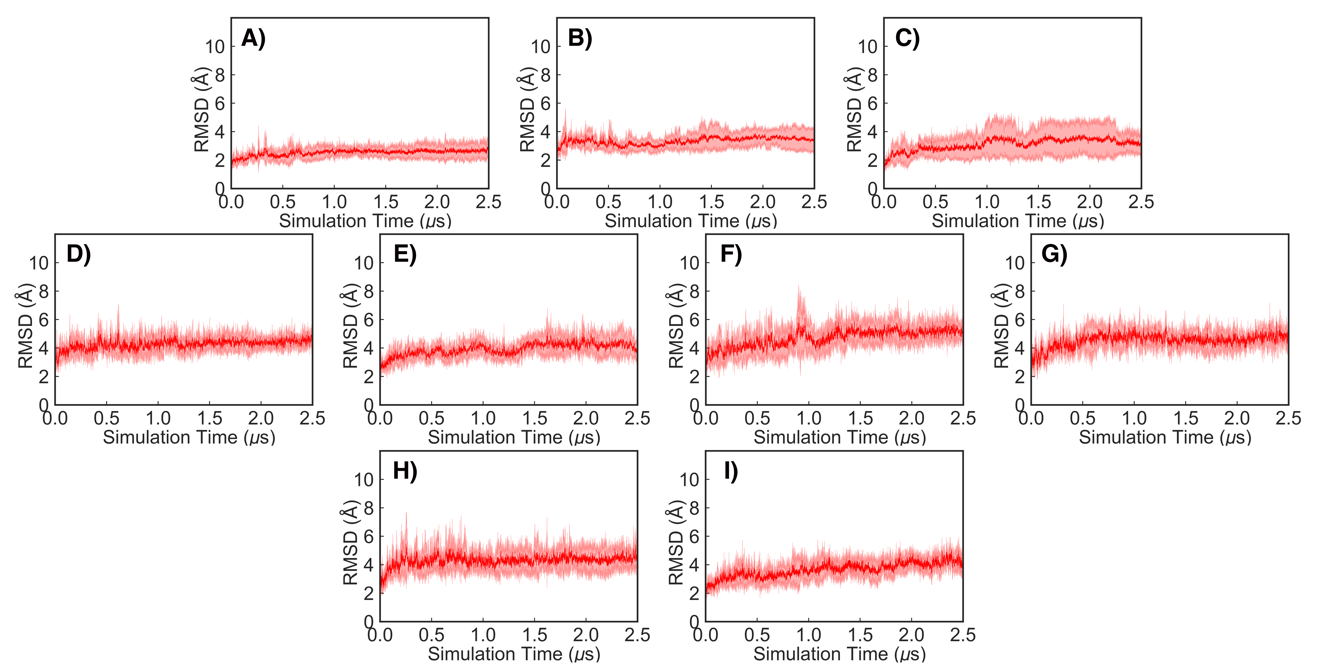


Root mean square deviation (RMSD, Å) of all protein backbone atoms of free MarA (A, B, C) I13C/M47C, I16C/M47C disulfide bridge variants, and I16C/M47C variant without the disulfide bridge, respectively; of all protein backbone atoms of MarA in complex with mar promoter (D, E, F, G) wild type, I16C/M47C disulfide bridge variant, I16C/M47C variant without the disulfide bridge and I16S/M47S double variant, respectively; as well as, of all protein backbone atoms of Rob in complex with the mar promoter (H, I) wild type and L10C/M41C disulfide bridge variant, respectively. RMSDs are calculated over 5 independent 2.5 μs molecular dynamics simulations, and relative to the initial constructs. The solid lines denote the average RMSD over all replicas, and the shaded lines show the standard deviations over the different individual replicas.

Fig. S9.

*
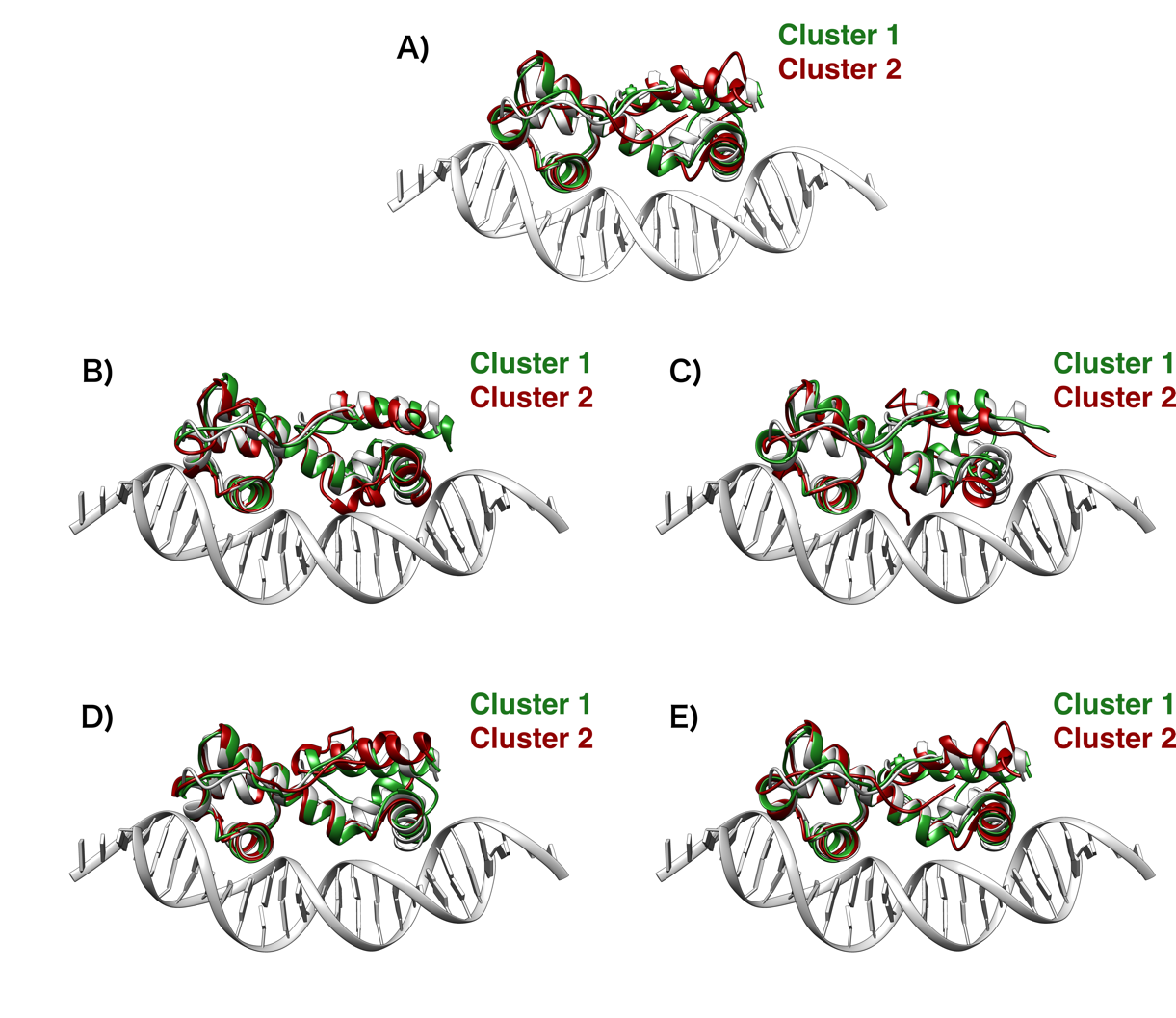
*
Centroids of the first two clusters obtained from hierarchical agglomerative clustering on simulations of free MarA, with root mean square deviation (RMSD) clustering performed over 5 independent 2.5 μs molecular dynamics simulations of (A) wild type, (B) I13C/M47C, (C) I16C/M47C and (D, E) I13C/M47C and I16C/M47C MarA without the disulfide bridge present, respectively. An overlay of MarA crystal structure bound to the DNA (PDB ID: 1BL0, ^1^) is shown in white. The clusters were extracted as indicated in the Methods section.

Fig. S10.

*
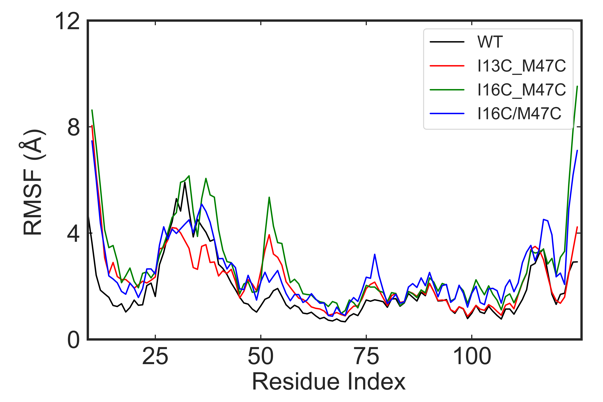
*

Root mean square fluctuations (RMSF, Å) of the Cα-atoms of DNA-free MarA variants considered in this work, calculated over five independent 2.5 μs MD simulations (Supplementary Materials Table S2).

Fig. S11.


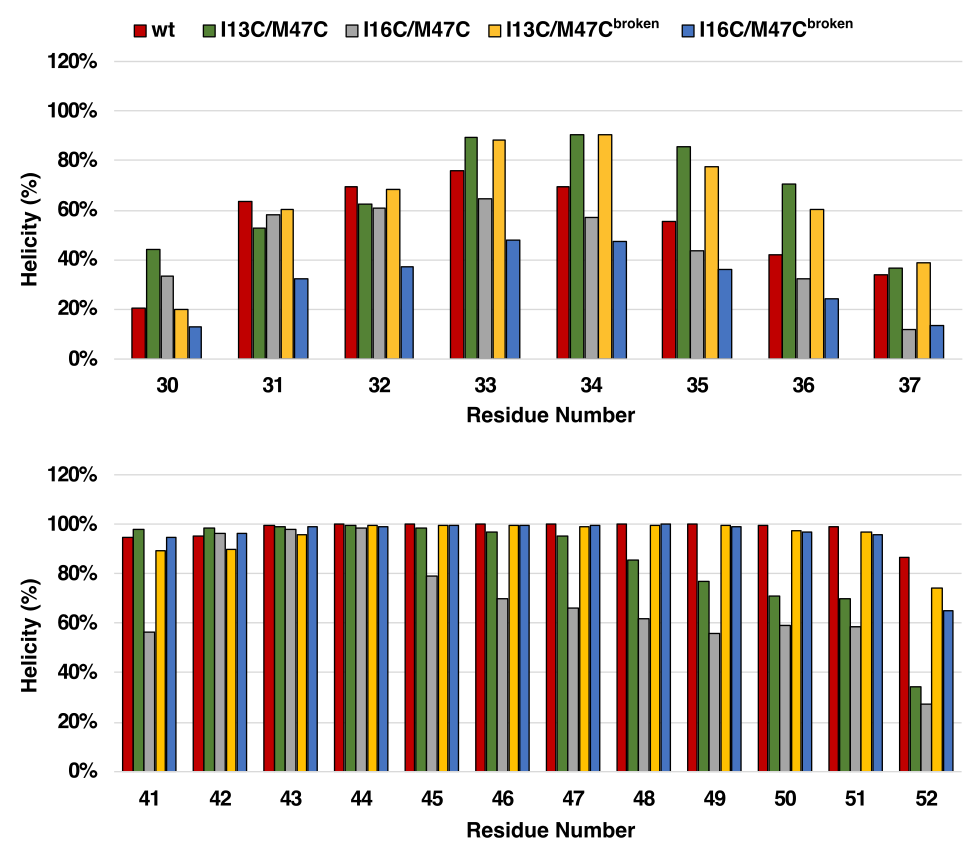


Helicity propensities for all residues belonging to the helix-turn-helix (HTH) motif encompassing helices H2 and H3 of free wild type MarA (red), the I13C/M47C double variant MarA with a disulfide bridge (green), the I16C/M47C double variant MarA with a disulfide bridge (grey) and the I13C/M47C and I16C/M47C double variants MarA with an artificially broken disulfide bridge to mimic the effect of adding DDT to the system (yellow and blue, respectively).

**Fig. S12.**


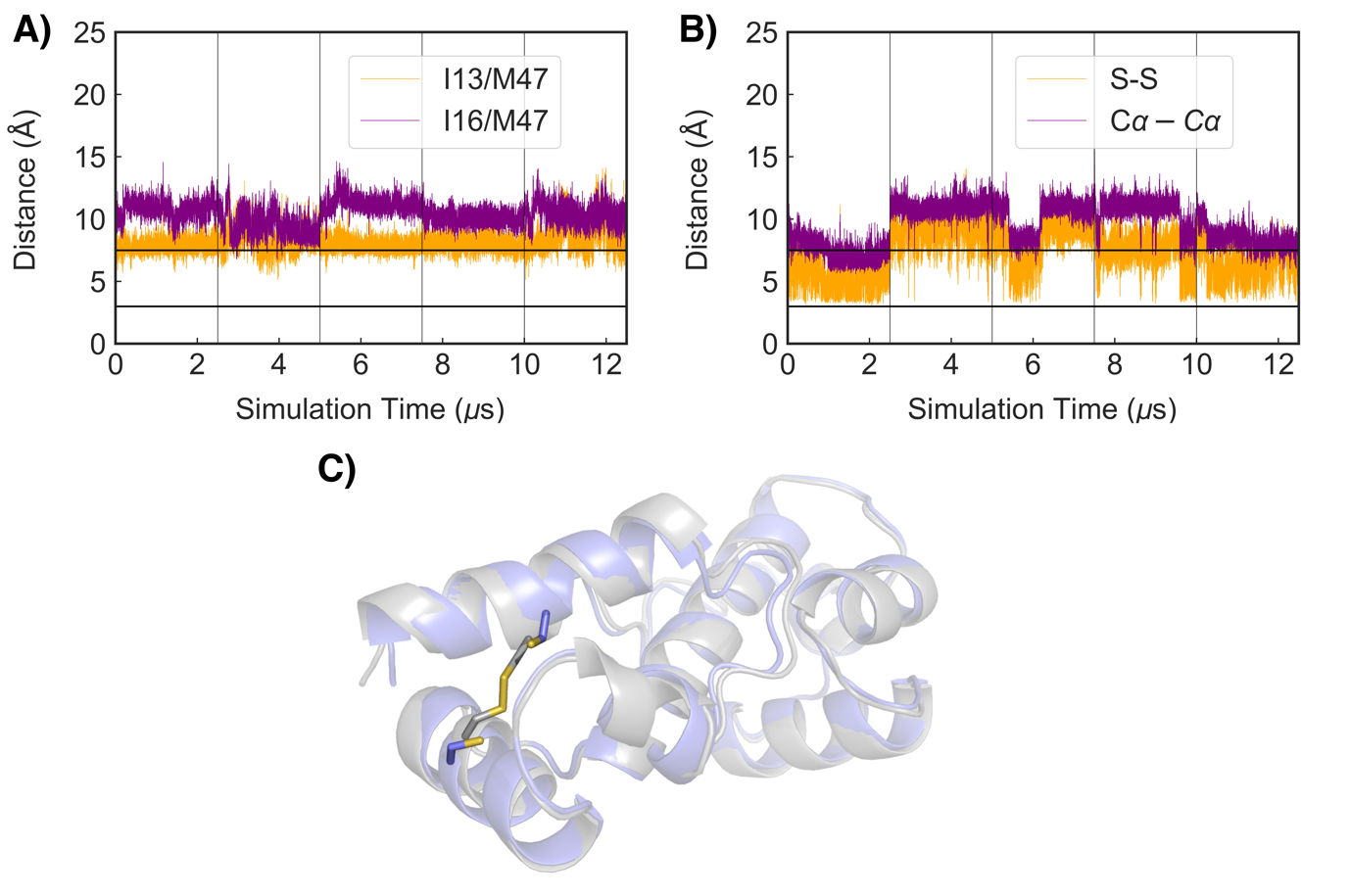


Time evolution of the distances between the C_α_ carbon atoms of (A) I16/M47 and I13/M47 in wild type MarA, and (B) time evolution of the distances between alpha carbons and sulphur atoms of I16C/M47C, where the disulfide bridge is not present because the cysteines are reduced to SH. (C) Overlay of the structures of I16C/M47C MarA before and after the disulfide bridge formation.

Fig. S13.


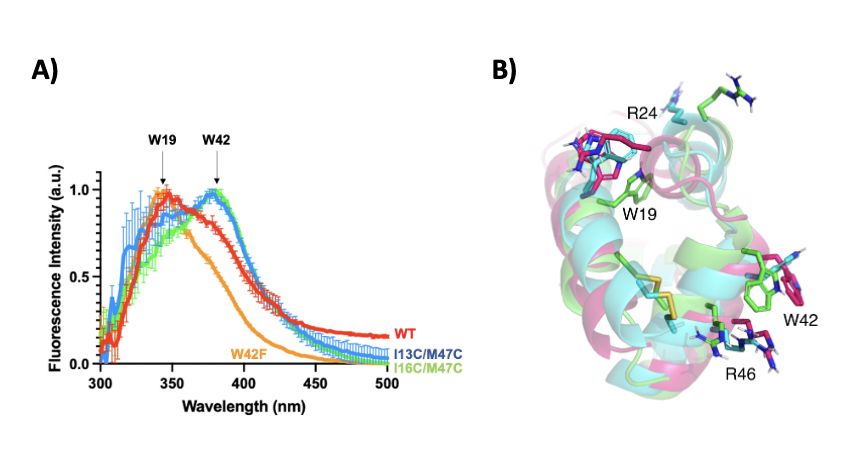


(A) Intrinsic fluorescence measurements of WT MarA, I13C/M47C and the I16C/M47C variants. The W42F variant was synthesised to distinguish between the signal generated by W19 (located in the N-terminal helix) and the one generated by W42 (located in the helix 3, which contact directly the DNA (Figure 1)). The I13C/M47C and I16C/M47C variants showed differences in the peak related to W19 (around 350 nm) but not related to W42 (around 380 nm). This suggests that the inability of I16C/M47C to bind DNA after disulfide bond formation does not relay on the distortion of the overall fold of the N-terminal domain, but in the immobilisation of the N-terminal helix. (B) Close-up showing the conformational changes of W19 and W42 of the centroids of the most populated cluster obtained from hierarchical agglomerative clustering on simulations of free MarA, with root mean square deviation (RMSD) clustering performed over 5 independent 2.5 μs moleculardynamics simulations of wild-type (magenta), I13C/M47C (blue) and I16C/M47C (green) variants.

Fig. S14.

*
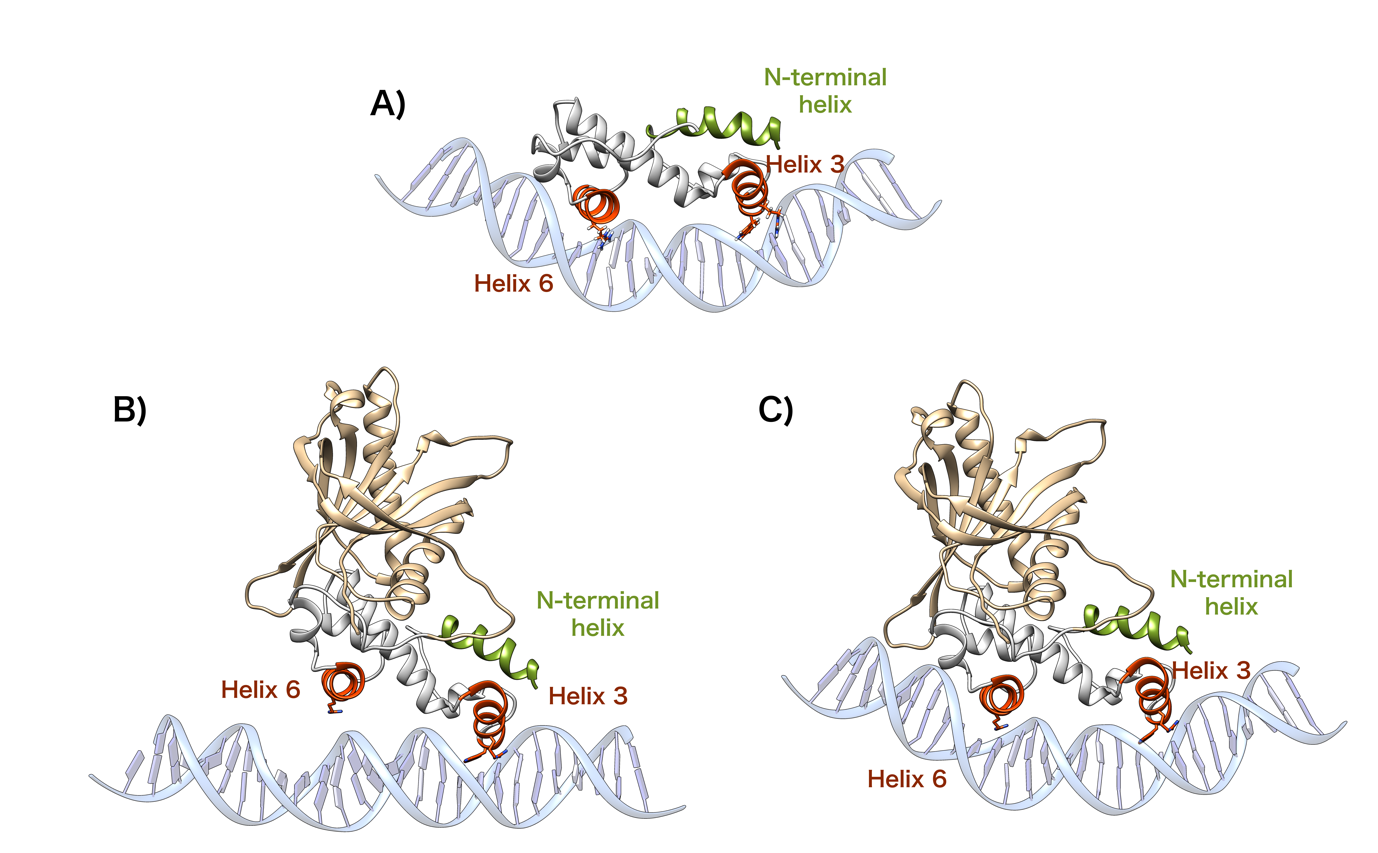
*

Starting structures used in our molecular dynamic simulations of wild type and variant complexes of **(A)** MarA-*mar*, **(B)** Rob- *mar* and **(C)** Rob- *mar* with bent DNA. Helices 3 and 6 are depicted in orange-red while the N-terminal helix, where the disulfide bridge between helix 3 is created, is shown in green. Residues displaying key interactions with the DNA nucleobases are shown as sticks.

Fig. S15.


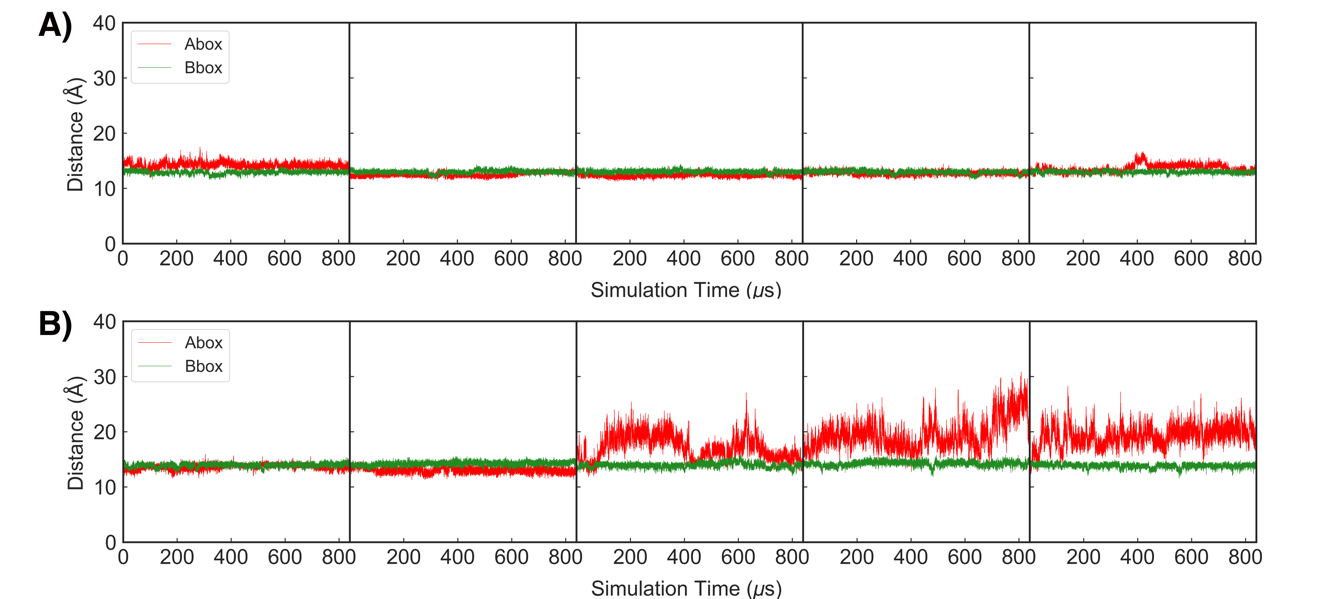


Time evolution of the distances between helices 3 and 6 of MarA, which are inserted inside the major groove of mar, and the base pairs at the A- and B-boxes, respectively, during 5 individual replicas of GaMD simulations of (A) the MarA(I13C/M47C)-mar complex, and (B) the MarA(I16C/M47C)-mar complex.

Fig. S16.


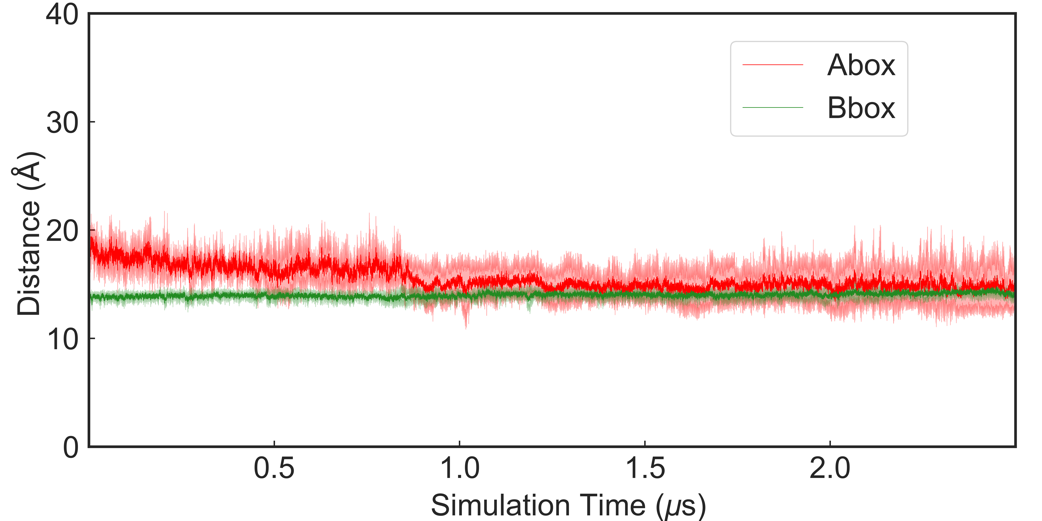


Time evolution of the distances between the helices inserted inside the major groove and the base pairs at the A- and B-boxes during 2.5 μs of conventional MD simulations of the MarA(I16S/M47S)-*mar* complex. Note that the starting point for these simulations was the A-box unbound MarA(I16C/M47C)-*mar* complex obtained from our GaMD simulations, where the corresponding disulfide bridge was broken and each cysteine was exchanged by a serine.

Fig. S17.


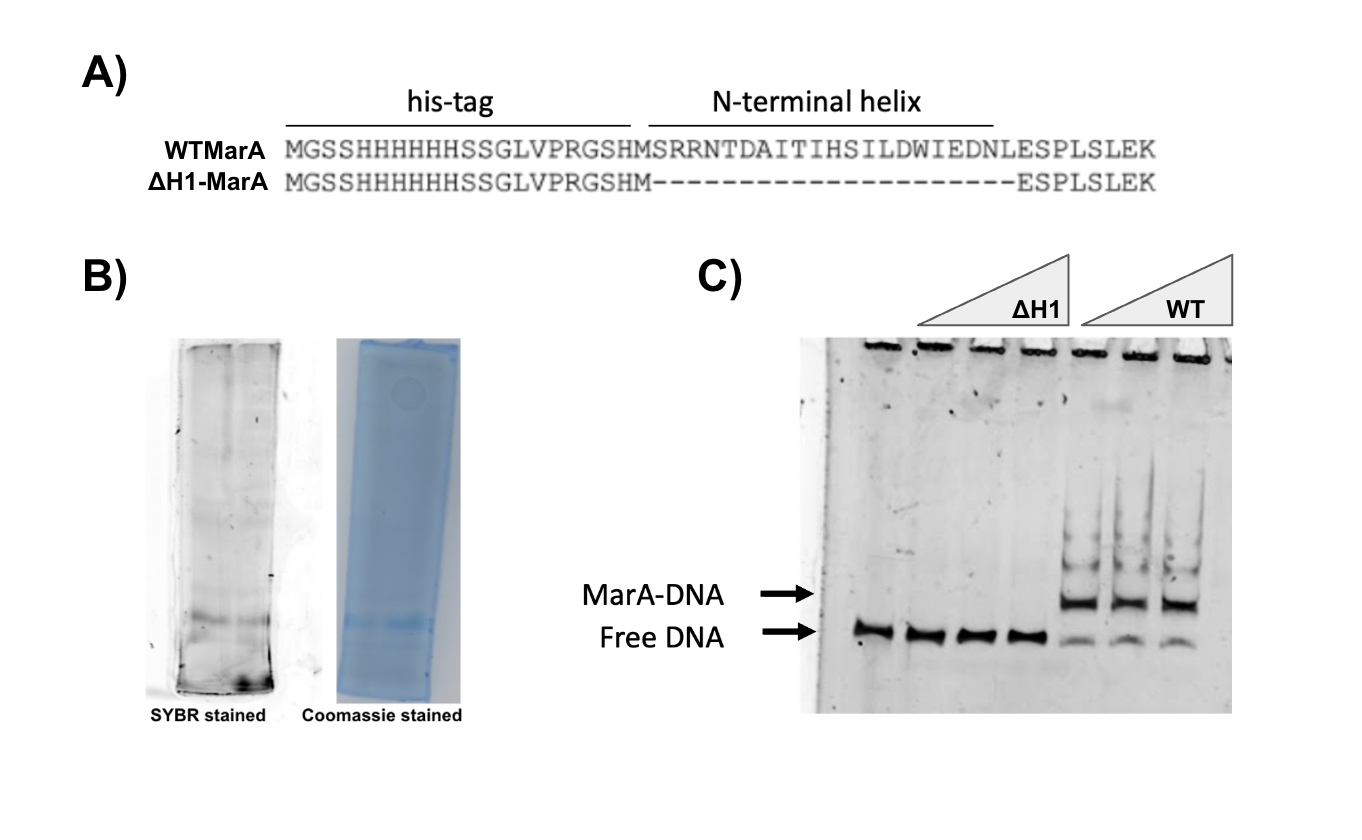


ΔH1-MarA. **(A)** Alignment of the His-tagged wild type (WT) MarA N-terminal region and ΔH1-MarA. The deleted amino acids and the His-tag that occupies their position can be observed. **(B)** Purified ΔH1-MarA in an acrylamide gel stained with SYBR green (DNA detection) and Coomassie (protein detection). Both DNA and protein are in the same band, suggesting their co-existence as a MarA-DNA complex. **(C)** EMSA with the WT MarA protein and the ΔH1-MarA. As expected, ΔH1-MarA was not able to bind the 200-bp DNA fragment containing the *marRAB* marbox.

Fig. S18.


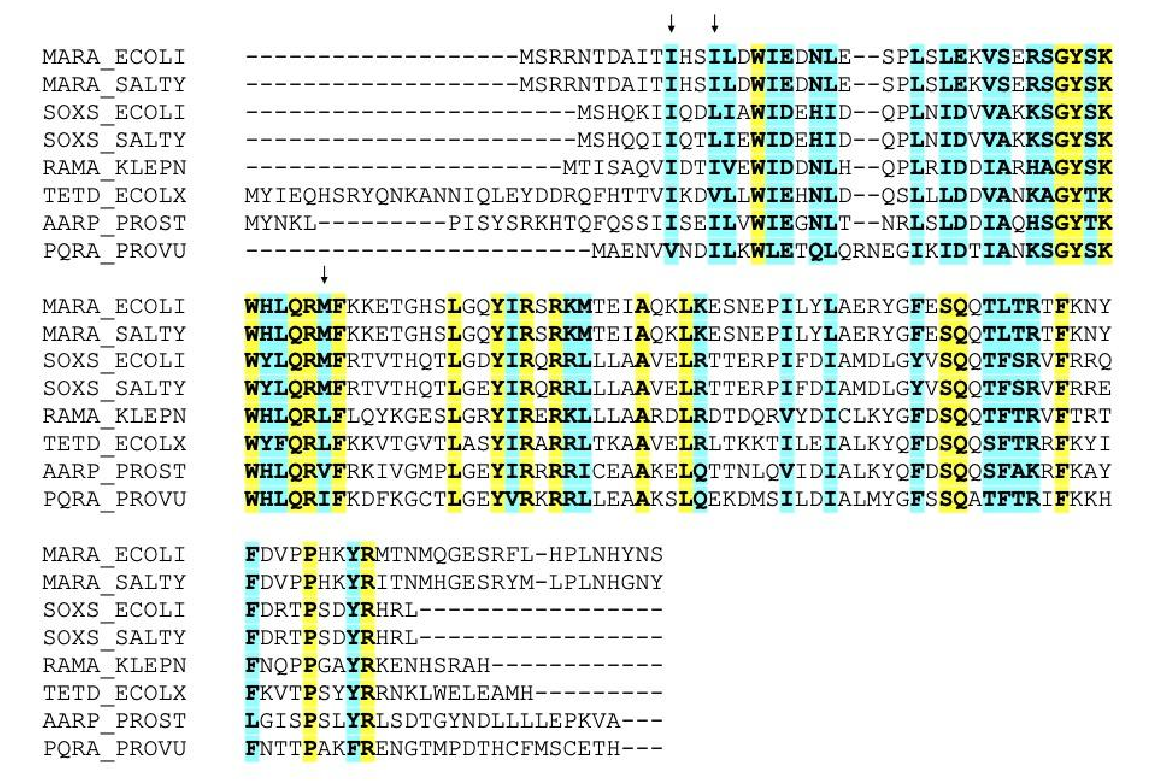

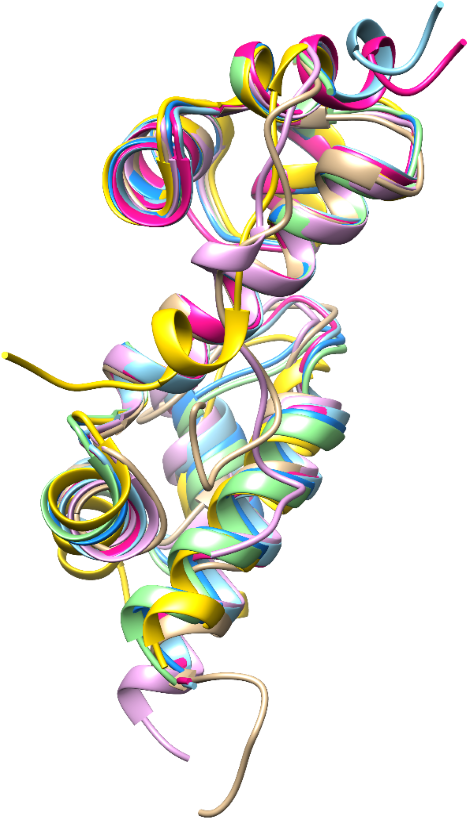


Comparison with other AraC/XylS family members consisting exclusively of the DNA binding domain (DBD). **(A)** Superposition of MarA, SoxS, RamA, TetD, Aarp, PqrA (structures predicted by AlphaFold with a high level of confidence (pLDDTs between 83 and 93) ^2,3^). **(B)** Alignment of the amino acid sequences showing the conservation of the key residues in this work. The conserved and similar residues are shadowed in yellow and cyan, respectively.

Fig. S19.


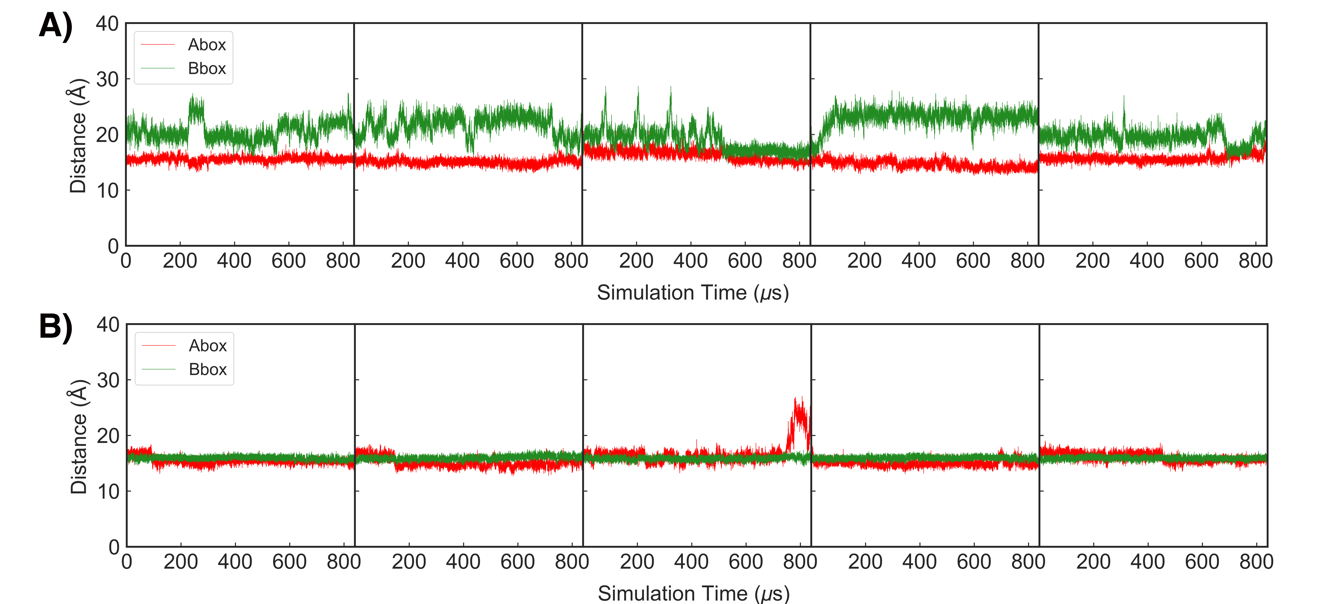


Time evolution of the distances between the helices 3 and 6 of the Rob(L10C/M41C) variant, which are inserted inside the major groove of mar, and the base pairs at the A- and B-boxes, respectively, during 5 individual replicas of GaMD simulations of the Rob(L10C/M41C)-mar complex. Simulations were initiated from (A) the crystallographic DNA binding mode and (B) the double bound DNA binding mode, with the DNA bent as in the MarA crystal structure (see the main text for details).

Table S1. Primers used in this work.

| Primer name | Primer sequence (5´- 3´) |
| --- | --- |
| **Cloning *Escherichia coli* *marA* and generation of the *marA* mutants** | |
| MarA_NdeI_F | GAGGTATGCATATGTCCAGACGCAATACTG |
| MarA_HindIII_R | CAGTGACGTTGTCAAGCTTTCAACTAGCTG |
| MarA_I13C_F | CTGACGCTATTACCTGTCATAGCATTTTGGAC |
| MarA_I13C_R | CAAAATGCTATGACAGGTAATAGCGTCAGTATTG |
| MarA_I16C_F | CCATTCATAGCTGTTTGGACTGGATCGAGG |
| MarA_I16C_R | GATCCAGTCCAAACAGCTATGAATGGTAATAG |
| MarA_M47C_F | CACCTGCAACGGTGTTTTAAAAAAGAAACC |
| MarA_M47C_R | CTTTTTTAAAACACCGTTGCAGGTGCC |
| MarA_S62C_F | CAATACATCCGCTGCCGTAAGATGAC |
| MarA_S62C_R | CTTACGGCAGCGGATGTATTGGC |
| MarA_L121C_F | GAATCGCGCTTTTGTCATCCATTAAATCATTAC |
| MarA_L121C_R | GATTTAATGGATGACAAAAGCGCGATTCGC |
| W42F_F | GGTTACTCTAAATTCCACCTGCAACGG |
| W42F_R | GTTGCAGGTGGAATTTAGAGTAACCTG |
| MarA_NdeI_deletionH1_F | CTGGATCGAGGACCATATGGAATCGCCACTGTC |
| **Amplification of the DNA fragments used in the EMSAs** | |
| *acrAB*_long_prom.FOR | GCTTTTGCAATCTCGCCCAGC |
| *acrAB*_long_prom.REV | GTCCGATTTCAAATTGGTCAATGG |
| *marRAB*_long_prom.FOR | GTTATCCTGTGTATCTGGGTTATCAGCG |
| *marRAB*_long_prom.REV | GTTGCCCTGGCAAGTAATTAGTTGC |
| non-specific_long.FOR | GACTGACGCTCAGGTGCGAAAG |
| non-specific_long.REV | CATGCTCCACCGCTTGTGCG |
| **30-bp DNA fragments used in the EMSAs** | |
| *acrAB*_short_prom.FOR | CTTCTTGTTTGGTTTTTCGTGCCATATGTTC |
| *acrAB*_short_prom.REV | GAACATATGGCACGAAAAACCAAACAAGAAG |
| *marRAB*_short_prom.FOR | GAACCGATTTAGCAAAACGTGGCATCGGTC |
| *marRAB*_short_prom.REV | GACCGATGCCACGTTTTGCTAAATCGGTTC |

Table S2. HPLC-ESI/MS for WT MarA in the absence (oxidised) and presence (reduced) of 1 mM DTT ^a^.

|  |  | WT oxidised  (expected mass 17205.56 Da) | | | WT reduced  (expected mass 17205.56 Da) | | |
| --- | --- | --- | --- | --- | --- | --- | --- |
|  | **[M+H]^+^**  **(expected)** | **[M+H]^+^**  **(observed)** | **observed mass (Da)** | **Δ mass**  **(Da)** | **[M+H]^+^**  **(observed)** | **observed mass (Da)** | **Δ mass**  **(Da)** |
| **MH9+** | 1912.73 | 1912.80 | 17206.20 | 0.64 | 1912.80 | 17206.20 | 0.64 |
| **MH10+** | 1721.56 | 1721.65 | 17206.50 | 0.94 | 1721.60 | 17206.00 | 0.44 |
| **MH11+** | 1565.15 | 1565.25 | 17206.75 | 1.19 | 1565.15 | 17205.65 | 0.09 |
| **MH12+** | 1434.80 | 1434.75 | 17205.00 | -0.56 | 1434.75 | 17205.00 | -0.56 |
| **MH13+** | 1324.51 | 1324.45 | 17204.85 | -0.71 | 1324.50 | 17205.50 | -0.06 |
| **MH14+** | 1229.97 | 1229.90 | 17204.60 | -0.96 | 1229.95 | 17205.30 | -0.26 |
| **MH15+** | 1148.04 | 1148.00 | 17205.00 | -0.56 | 1148.00 | 17205.00 | -0.56 |
| **MH17+** | 1013.09 | 1013.05 | 17204.85 | -0.71 | 1013.05 | 17204.85 | -0.71 |
| **MH19+** | 906.56 | 906.50 | 17204.50 | -1.06 | 906.45 | 17203.55 | -2.01 |
| **MH22+** | 783.08 | 783.10 | 17206.20 | 0.64 | 783.00 | 17204.00 | -1.56 |
| **MH24+** | 717.90 | 717.90 | 17205.60 | 0.04 |  |  |  |
| **MH26+** | 662.77 | 662.80 | 17206.80 | +1.24 | 662.75 | 17205.50 | -0.06 |
| **MH30+** | 574.52 |  |  |  | 574.55 | 17206.50 | 0.94 |

^a^ The expected peaks and expected mass are similar since WT MarA lacks any cysteine in the amino acid sequence. The expected monoisotopic molecular mass values (*[M+H]^+^) were calculated by using the GPMAW software (see the material and methods section). The observed mass was calculated by using the observed* (*[M+H]^+^). Δ mass indicates the variation of mass between the observed and the expected molecular mass.*

**Table S3.** *HPLC-*ESI*/*MS for I13C/M47C in the absence (oxidised) of 1 mM DTT ^a^.

|  | I13C/M47C oxidised  (expected mass 17165.44 Da) | | | |  |
| --- | --- | --- | --- | --- | --- |
|  | **[M+H]^+^**  **(expected)** | **[M+H]^+^**  **(observed)** | **observed mass (Da)** | **Δ mass**  **(Da)** | **paired/not paired** |
| **MH9^+^** | 1908.27 | 1908.20 | 17164.80 | -0.64 | -S-S- |
| **MH10^+^** | 1717.55 | 1717.60 | 17166.0 | +0.56 | -S-S- |
| **MH11^+^** | 1561.50 | 1561.70 | 17167.70 | +2.26 | -SH    HS- |
| **MH12^+^** | 1431.46 | 1431.90 | 17170.80 | +5.36 | N.I. |
| **MH13^+^** | 1321.42 | 1321.60 | 17167.80 | +2.36 | -SH    HS- |
| **MH14^+^** | 1227.11 | 1227.25 | 17167.50 | +2.06 | -SH    HS- |
| **MH16^+^** | 1073.84 | 1073.65 | 17162.40 | -3.04 | N.I. |
| **MH17^+^** | 1010.74 | 1010.70 | 17164.90 | -0.54 | -S-S- |
| **MH18^+^** | 954.64 | 954.70 | 17166.60 | +1.16 | N.I. |
| **MH22^+^** | 781.25 | 781.25 | 17165.50 | +0.06 | -S-S- |
| **MH23^+^** | 747.33 | 747.35 | 17166.05 | +0.61 | -S-S- |

^a^ The expected monoisotopic molecular mass values (*[*M+H]^+^) were calculated by using the GPMAW software (see the material and methods section). The observed mass was calculated by using the observed ([M+H]^+^). Δ mass indicates the variation of mass between the observed and the expected molecular mass. N.I. non-identified specie.

**Table S4*.*** HPLC-ESI*/*MS for I13C/M47C in the presence (reduced) of 1 mM DTT ^a^.

|  | I13C/M47C reduced  (expected mass 17167.46 Da) | | | |  |
| --- | --- | --- | --- | --- | --- |
|  | **[M+H]^+^**  **(expected)** | **[M+H]^+^**  **(observed)** | **observed mass (Da)** | **Δ mass**  **(Da)** | **paired/not paired** |
| **MH9^+^** | 1908.50 | 1908.55 | 17167.95 | +0.49 | -SH    HS- |
| **MH10^+^** | 1717.75 | 1717.70 | 17167.0 | -0.46 | -SH    HS- |
| **MH11^+^** | 1561.68 | 1561.40 | 17164.40 | -3.06 | N.I. |
| **MH12^+^** | 1431.63 | 1431.20 | 17162.40 | -5.06 | N.I. |
| **MH13^+^** | 1321.58 | 1321.40 | 17165.20 | -2.26 | -S-S- |
| **MH14^+^** | 1227.25 | 1227.45 | 17170.30 | +2.84 | N.I. |
| **MH16^+^** | 1073.97 | 1073.90 | 17166.40 | -1.06 | N.I. |
| **MH18^+^** | 954.75 | 954.70 | 17166.60 | -0.86 | -SH HS- |
| **MH22^+^** | 781.34 | 781.35 | 17167.70 | +0.24 | -SH    HS- |
| **MH24^+^** | 716.31 | 716.45 | 17170.80 | +3.34 | N.I. |
| **MH27^+^** | 636.83 | 636.80 | 17166.60 | -0.86 | -SH HS- |

^a^ The expected monoisotopic molecular mass values (*[M+H]^+^) we*re calculated by using the GPMAW software (see the material and methods section). The observed mass was calculated by using the observed ([M+H]^+^). Δ mass indicates the variation of mass between the observed and the expected molecular mass. N.I. non-identified specie.

**Table S5.** HPLC-ESI*/*MS for I16C/M47C in the absence (oxidised) of 1 mM DTT^a^.

|  | I16C/M47C oxidised  (expected mass 17165.44 Da) | | | |  |  |
| --- | --- | --- | --- | --- | --- | --- |
|  | **[M+H]^+^**  **(expected)** | **[M+H]^+^**  **(observed)** | **observed mass (Da)** | **Δ mass**  **(Da)** | **paired/not paired** |  |
| **MH9^+^** | 1908.27 | 1908.50 | 17167.50 | +2.10 | -SH    HS- |  |
| **MH10^+^** | 1717.55 | 1718.0 | 17170.0 | +4.60 | N.I. |  |
| **MH11^+^** | 1561.50 | 1561.90 | 17169.90 | +4.50 | N.I. |  |
| **MH12^+^** | 1431.46 | 1431.50 | 17166.0 | +0.60 | -S-S- |  |
| **MH13^+^** | 1321.42 | 1322.90 | 17174.70 | +19.30 | N.I. |  |
| **MH14^+^** | 1227.11 | 1227.15 | 17166.10 | +0.70 | -S-S- |  |
| **MH15^+^** | 1145.37 | 1145.35 | 17165.25 | -0.15 | -S-S- |  |
| **MH17^+^** | 1010.74 | 1010.65 | 17164.05 | -1.35 | N.I. |  |
| **MH18^+^** | 954.64 | 954.75 | 17167.50 | +2.10 | -SH    HS- |  |
| **MH21^+^** | 818.40 | 818.40 | 17165.40 | 0 | -S-S- |  |
| **MH23^+^** | 747.33 | 747.40 | 17167.20 | +1.80 | -SH    HS- |  |

^a^ The expected monoisotopic molecular mass values ([M+H]^+^) were calculated by using the GPMAW software (see the material and methods section). The observed mass was calculated by using the observed ([M+H]^+^). Δ mass indicates the variation of mass between the observed and the expected molecular mass. N.I. non-identified specie.

**Table S6.** HPLC-ESI*/*MS for I16C/M47C in the presence (reduced) of 1 mM DTT ^a^.

|  | I16C/M47C reduced  (expected mass 17167.46 Da) | | | |  |  |
| --- | --- | --- | --- | --- | --- | --- |
|  | **[M+H]^+^**  **(expected)** | **[M+H]^+^**  **(observed)** | **observed mass (Da)** | **Δ mass**  **(Da)** | **paired/not paired** |  |
| **MH9^+^** | 1908.50 | 1908.50 | 17167.50 | +0.04 | -SH    HS- |  |
| **MH10^+^** | 1717.75 | 1717.95 | 17169.50 | +2.04 | N.I. |  |
| **MH11^+^** | 1561.68 | 1562.10 | 17172.10 | +4.64 | N.I. |  |
| **MH12^+^** | 1431.63 | 1431.60 | 17167.20 | -0.26 | -SH    HS- |  |
| **MH13^+^** | 1321.58 | 1322.90 | 17184.70 | +17.24 | N.I. |  |
| **MH14^+^** | 1227.25 | 1227.20 | 17166.80 | -0.66 | -SH    HS- |  |
| **MH15^+^** | 1145.50 | 1145.35 | 17165.25 | -2.21 | -S-S- |  |
| **MH17^+^** | 1010.86 | 1010.70 | 17164.90 | -2.56 | -S-S- |  |
| **MH18^+^** | 954.75 | 954.80 | 17168.40 | +0.94 | -SH    HS- |  |
| **MH21^+^** | 818.50 | 818.35 | 17164.35 | -3.11 | N.I. |  |

^a^ The expected monoisotopic molecular mass values ([M+H]^+^) were calculated by using the GPMAW software (see the material and methods section). The observed mass was calculated by using the observed ([M+H]^+^). Δ mass indicates the variation of mass between the observed and the expected molecular mass. N.I. non-identified specie.

**Table S7.** Summary of all simulations performed in this work.^a^

| **Systems** | | **Individual Simulations (μs)** | **Total Simulation Time (μs)** |
| --- | --- | --- | --- |
| **Conventional Molecular Dynamics simulations** | | | |
| Free MarA | I13C/M47C disulfide bridge | 5 × 2.5 | 12.5 |
|  | I16C/M47C  disulfide bridge | 5 × 2.5 | 12.5 |
|  | I13C/M47C | 5 × 2.5 | 12.5 |
|  | I16C/M47C | 5 × 2.5 | 12.5 |
| MarA-*mar* complexes | I13C/M47C disulfide bridge | 5 × 2.5 | 12.5 |
|  | I16C/M47C  disulfide bridge | 5 × 2.5 | 12.5 |
| MarA-*mar* complexes from unbound structure | WT | 5 × 2.5 | 12.5 |
|  | I16C/M47C  disulfide bridge | 5 × 2.5 | 12.5 |
|  | I16C/M47C | 5 × 2.5 | 12.5 |
|  | I16S/M47S | 5 × 2.5 | 12.5 |
| Rob-*mar* complexes from unbound structure | WT | 5 × 2.5 | 12.5 |
|  | I16C/M47C  disulfide bridge | 5 × 2.5 | 12.5 |
| **Gaussian Accelerated Molecular dynamics simulations** | | | |
| MarA-*mar* complexes | I13C/M47C disulfide bridge | 5 × 0.804 | 4.02 |
|  | I16C/M47C  disulfide bridge | 5 × 0.804 | 4.02 |
| Rob-*mar* complexes | I16C/M47C  disulfide bridge^b^ | 5 × 0.804 | 4.02 |
|  | I16C/M47C  disulfide bridge^c^ | 5 × 0.804 | 4.02 |
| Total simulation time | | | 166.08 |

^a^ Summary of the number of individual trajectories studied per system, as well as the cumulative simulation time over all trajectories per system, and over all systems in total. All simulation times are given in μs. ^b^ Simulations from single B-box bound structure mimicking the crystallographic conformation (PDB ID: 1D5Y, ^4^). ^c^ Simulations initiated from the double bound (A- and B-boxes) Rob structure.

**SI References**

1. Rhee S, Martin RG, Rosner JL, Davies DR (1998) A novel DNA-binding motif in MarA: the first structure for an AraC family transcriptional activator. Proc. Natl. Acad. Sci. U. S. A. 95:10413–10418.

2. Jumper J, Evans R, Pritzel A, Green T, Figurnov M, Ronneberger O, Tunyasuvunakool K, Bates R, Žídek A, Potapenko A, et al. (2021) Highly accurate protein structure prediction with AlphaFold. Nature 596:583–589.

3. Varadi M, Anyango S, Deshpande M, Nair S, Natassia C, Yordanova G, Yuan D, Stroe O, Wood G, Laydon A, et al. (2022) AlphaFold Protein Structure Database: massively expanding the structural coverage of protein-sequence space with high-accuracy models. Nucleic Acids Res. 50:D439–D444.

4. Kwon HJ, Bennik MH, Demple B, Ellenberger T (2000) Crystal structure of the Escherichia coli Rob transcription factor in complex with DNA. Nat. Struct. Biol. 7:424–430.
